# Habitat shapes the lipidome of the tropical photosynthetic sea slug *Elysia crispata*

**DOI:** 10.1007/s42995-025-00281-1

**Published:** 2025-04-07

**Authors:** Felisa Rey, Xochitl Guadalupe Vital, Sónia Cruz, Tânia Melo, Diana Lopes, Ricardo Calado, Nuno Simões, Maite Mascaró, Maria Rosário Domingues

**Affiliations:** 1 Centre for Environmental and Marine Studies (CESAM), Department of Chemistry, Campus Universitário de Santiago, University of Aveiro, 3810-193 Aveiro, Portugal; 2 Mass Spectrometry Centre & LAQV-REQUIMTE, Department of Chemistry, Campus Universitário de Santiago, University of Aveiro, 3810-193 Aveiro, Portugal; 3 Posgrado en Ciencias Biológicas, Unidad de Posgrado, Edificio D, 1° Piso, Circuito de Posgrados, Ciudad Universitaria, Alcaldía Coyoacán, 04510 Ciudad de Mexico, México; 4 UMDI-Sisal, Facultad de Ciencias, Universidad Nacional Autónoma de México, Puerto de Abrigo S/N, 97356 Sisal, Mexico; 5 ECOMARE-Laboratory for Innovation and Sustainability of Marine Biological Resources, CESAM, Department of Biology, University of Aveiro, Campus Universitário de Santiago, 3810-193 Aveiro, Portugal; 6 International Chair for Coastal and Marine Studies, Harte Research Institute for Gulf of Mexico Studies, Texas A and M University-Corpus Christi, Corpus Christi, TX 78412 USA; 7 Laboratorio Nacional de Resiliencia Costera (LANRESC), Laboratorios Nacionales, CONACYT, 97356 Sisal, Mexico

**Keywords:** Kleptoplasty, Lipidomics, Marine invertebrates, Marine lipids, Photosynthetic animals, Polar lipids

## Abstract

Sacoglossan sea slugs have attracted considerable scientific attention due to their capacity to retain functional macroalgal chloroplasts inside their cells. This endosymbiotic association is nutritionally relevant for these organisms and represents an interesting research issue for biotechnological applications. The Caribbean species *Elysia crispata* can integrate chloroplasts from different macroalgal species. The lipidome of chloroplasts includes lipid classes unique to these photosynthetic organelles. Specialized lipids, such as the glycolipids MGDG, DGDG, and SQDG, are essential for maintaining the integrity of both the thylakoid membranes and the overall chloroplast membrane structure. Additionally, lipids are a diverse group of biomolecules playing essential roles at nutritional and physiological levels. A combined approach using LC–HR-MS and MS/MS was employed to determine the polar lipid profile of the photosynthetic sea slug *E. crispata* from two habitats in the north-western tropical Atlantic (Sistema Arrecifal Veracruzano and Mahahual) and two different feeding conditions (fed and after 1 week of starvation). Significant differences were identified in the abundance of structural and signalling phospholipids (PC, PI, PG, PS, CL) suggesting different nutritional states between populations. The composition of glycolipids demonstrated a clear separation by habitat, but not by feeding conditions. The lower abundance of glycolipids in the Mahahual samples suggests a lower density of chloroplasts in their tissues compared to Veracruz individuals. These results corroborate that 1 week of starvation is insufficient to initiate the degradation of plastid membranes. This study confirms the advantages of using lipidomics as a tool to enhance our knowledge of the ecology of marine invertebrates.

**Supplementary Information:**

The online version contains supplementary material available at 10.1007/s42995-025-00281-1.

## Introduction

Sacoglossan sea slugs live at the interface between two spheres of the marine realm, animal fauna and algal flora. This group of marine invertebrates includes several species that have a lifelong association with stolen chloroplasts provided through their diet. In general, sacoglossan sea slugs are very selective in their food sources, feeding on a reduced number of large siphonaceous macroalgae, although not all sea slug species are strictly monophagous (Curtis et al. 2015; Händeler et al. 2010; Maeda et al. 2012). After ingesting the content of the algal cells, they digest the entire cell content except for the chloroplasts, which are engulfed by the digestive system of the sea slug cells and remain functional for variable time periods (from hours to months) (Chihara et al. 2020; Händeler et al. 2009; Laetz and Wägele 2017; Trench et al. 1969). The common view is that these species are mixotrophic organisms obtaining organic carbon via both heterotrophic and autotrophic pathways (Cartaxana et al. 2019, 2021; Cruz et al. 2020; Maeda et al. 2012). Despite evidence that sequestered chloroplasts' (known as kleptoplasts) photosynthesis supports survival of sea slugs during periods of food scarcity in several species, there are opposing views that cannot be ignored (see latest review by Cruz and Cartaxana 2022), and references therein). However, the retention time of functional kleptoplasts inside animal cells is conditioned by several factors, such as the chloroplasts’ algal donor, the sea slug species, its life cycle stage or even light conditions (Cartaxana et al. 2023; Cruz et al. 2013; Laetz et al. 2017b). Two recent studies used isotopic labelling in two sea slug species with long-term kleptoplasty to understand metabolite transfer from kleptoplasts to the host animal cells (Cartaxana et al. 2021; Cruz et al. 2020). The use of compound-specific isotope analysis (CSIA) and high-resolution secondary ion mass spectrometry (NanoSIMS) allowed the identification of the translocation of photosynthesis-derived primary metabolites from functional kleptoplasts to kleptoplast-free reproductive organs (e.g. the albumen gland and gonadal follicles) of the animal host (Cartaxana et al. 2021; Cruz et al. 2020). Additionally, the use of isotopic labelling allowed the elucidation of the synthesis pathway of polyunsaturated fatty acids (PUFA) (Cartaxana et al. 2021). These results paved the way for further studies on the role that lipids may play in kleptoplasty.

Lipids serve as the primary energy source in marine environments, offering at least two-thirds more energy per gram than proteins or carbohydrates (Parrish 2013). After ingestion, lipids are decomposed to acylglycerols and fatty acids that can be used to biosynthesize new acyl lipids. In sacoglossan sea slugs, functional kleptoplasts are maintained structurally stable, including the exclusive lipids found in chloroplast membranes, namely glycolipids (Rey et al. 2017). This intricate lipidome equips photosynthetic sea slugs with a diverse array of lipids, merging unique compounds from both animal and algal tissues, alongside shared lipids present in both organisms (Rey et al. 2020, 2023). Polar lipids, including phospholipids, glycolipids, betaine lipids and sphingolipids, constitute the fundamental structures of biological membranes. Phospholipids, such as phosphatidylcholines (PC) and phosphatidylethanolamines (PE), are prevalent in both animal and algal tissues, serving as key components of membranes. In algae, most phospholipids are part of extraplastidial membranes, except for phosphatidylglycerols (PG), which are primarily located in thylakoid membranes (Lopes et al. 2023). Glycolipids are exclusive to chloroplast membranes, while betaine lipids can be synthesized by algae, bacteria, fungi and lower land plants (Cañavate et al. 2016; Sato 1992;). The presence of glycolipids (monogalactosyl diacylglycerol [MGDG], digalactosyl diacylglycerol, [DGDG], sulphoquinovosyl diacylglycerol [SQDG] and diacylglyceryl glucuronide, [DGGA]) and betaine lipids (diacylglyceryl-*N*,*N*,*N*-trimethyl homoserine, DGTS) in sea slugs’ lipidome originates from sequestered chloroplasts. The predominant form of glycolipids is in their diacyl form, with an increase in their lyso forms (MGMG and DGMG) often linked to the activity of galactolipases (Hashiro et al. 2018), a process likely associated with the degradation of chloroplast membranes. Within the sphingolipid category, ceramide aminoethylphosphonates (CAEP) are unique to animal tissues and are extensively characterized in marine invertebrates, such as molluscs and cnidarians (Imbs et al. 2021; Wang et al. 2020). Therefore, a deeper investigation of the different lipid classes found in sea slug tissues can provide valuable insight into how these photosynthetic organisms adapt to variations in external food sources.

Most sacoglossan species feed on and retain chloroplasts from a limited number of closely related macroalgae species (Curtis et al. 2015; Händeler et al. 2009). *Elysia crispata* Mörch, 1863 is a sacoglossan sea slug species naturally distributed in the Gulf of Mexico and the Caribbean Sea (Jiménez et al. 2022; WoRMS Editorial Board 2024). *Elysia crispata* is known for its adaptability to different dietary scenarios, using a range of different macroalgae species available in its natural habitat as food sources (see supplementary Table S1 in Vital et al. 2021) and retaining chloroplasts from several species (Cartaxana et al. 2023; Middlebrooks et al. 2019). Most of the chloroplasts identified in *E. crispata* cells belong to the algal order Bryopsidales, although some Dasycladales species (see supplementary Table S1 in Vital et al. 2021; Cartaxana et al. 2023; Middlebrooks et al. 2019) have also been identified as the source of stolen chloroplasts (Middlebrooks et al. 2019).

Photosynthetic animals have attracted attention due to their potential in biotechnological applications (Torres et al. 2020). Lipidomic studies provide an in-depth understanding of lipid metabolism of photosynthetic sea slugs and expand our knowledge of lipid biochemistry and ecology. The present study used a lipidomics approach to better understand the influence of different habitats, and putative different chloroplast sources, on the polar lipid composition of *E. crispata*. The locations selected are from different regions, featuring contrasting biogeochemical processes (Carrillo et al. 2007), Verde reef in Sistema Arrecifal Veracruzano (Veracruz) and Mahahual. Veracruz is a habitat in southern Gulf of Mexico that has seasonal variations in salinity, temperature and turbidity, related to the discharges of nearby rivers and winter winds (Mateos-Jasso et al. 2012), while Mahahual in the Caribbean is influenced by submarine groundwater discharges, with almost no transportation of suspended sediments but with an important contribution of nutrients (mainly nitrogen and phosphorus) from inland (Camacho-Cruz et al. 2022; Hernández-Terrones et al. 2011; Null et al. 2014). In addition, massive drifts of *Sargassum* spp. have arrived in recent years, creating decreases in dissolved oxygen concentration water quality and an increase in ammonium in the coastal areas with high densities of this macroalga (Rodríguez-Muñoz et al. 2021). The trophic structure of herbivore invertebrates can also be affected by these events, as shown by a high consumption of *Halimeda* by the sea urchin *Diadema antillarum*, possibly related to an increased availability of this algae on this coral reef (Cabanillas-Terán et al. 2019). Also, a higher macroalgal coverage has been recorded in Mahahual compared to coral reefs in southern Gulf of Mexico, with substrate conditions promoting the development of multiple Chlorophyceae species (Garduño-Solórzano et al. 2005; Palomino-Alvarez et al. 2021).

It was hypothesized that different habitats provide different food abundance and sources, different light environments, together with other environmental parameters, resulting in differing physiological states of the photosynthetic sea slug *E. crispata.* Therefore, the objective of this study was to investigate the effect of habitat on the lipidome of *E. crispata*, with the aim of inferring its nutritional status. To achieve this objective, specimens were sampled from two different native habitats, Veracruz (Gulf of Mexico) and Mahahual (Caribbean). These specimens were analysed both in their wild state and after 1 week of starvation to assess changes in their nutritional status.

## Materials and methods

### Sampling

**Fig. 1:**
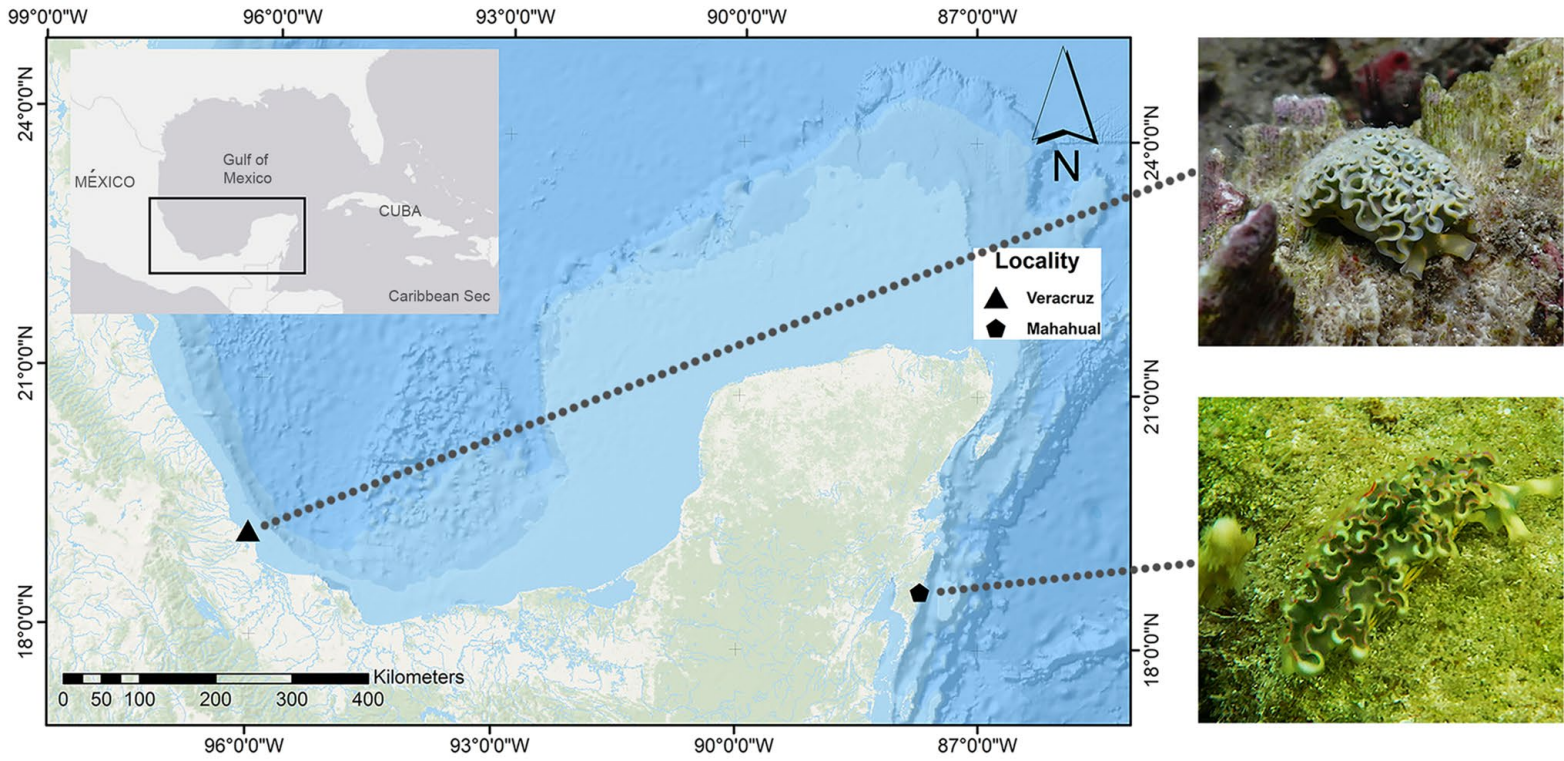
Coral reefs where *Elysia crispata* specimens were sampled in southern Gulf of Mexico (Veracruz) and the Mexican Caribbean (Mahahual) and specimens from each sampling area

Two different habitats were selected for sampling: Mahahual (Quintana Roo, México, 18° 41′ 10.62″ N, 87°42′ 59.30″ W) and Verde reef from Sistema Arrecifal Veracruzano (Veracruz, México 19° 12′ 11.90″ N, 96° 4′ 19.90″ W) in March and June 2020, respectively (Fig. 1). Collecting was conducted under a permit issued by SAGARPA (PPF/DGOPA-082/19). Animals measuring at least 50 mm in total length were collected from hard substrates at < 3 m depth. Immediately after sampling, seven specimens of *E. crispata* from each habitat were flash-frozen in liquid nitrogen and stored at − 80 °C before lyophilization. Additionally, specimens of *E. crispata* collected on these habitats were transported to the laboratory within 6 h in aerated seawater collected from the sampling sites. Animals were starved for a whole week (7 days) in the laboratory under constant conditions of salinity (35), temperature (26 °C) and light intensity (approximately, 60 µmol photons m^2^ s^−1^) in an aquarium with a volume of 14 L. The purpose of this starvation period was to empty the gut, ensuring that the lipidome analysed in the starved samples was not influenced by diet. Organisms were then flash-frozen in liquid nitrogen and stored at – 80 °C before lyophilization. Dry weight (DW) was measured in freeze-dried samples.

Seven samples of *E. crispata* fed and seven samples of *E. crispata* starved were obtained from Veracruz, and seven samples of *E. crispata* fed and seven samples of *E. crispata* starved were also collected from Mahahual. Seven samples × two feeding conditions × two habitats with a total of 28 sea slug samples. Fed specimens were those immediately frozen after collection, and it was assumed that they had eaten on the day they were collected.

### Lipid extraction

The lipid extract were obtained using the Bligh and Dyer protocol (Bligh and Dyer 1959). Freeze-dried samples were individually crushed with a pestle in a glass tube and mixed with 3750 µL methanol/dichloromethane (2:1, v:v). After vigorous homogenization and a short sonication step (1 min) (Bandelin, Sonorex, RK 100), the samples were incubated on ice for 30 min on a rocking platform shaker (Stuart Scientific SSL2) and centrifuged at 568×*g* for 10 min (Centurion Scientific Pro Analytical C4000R with a rotor BRK5324) at room temperature to collect the organic phase, which contains the lipids, in a glass tube. The biomass was re-extracted using an additional volume of 3750 µL methanol/dichloromethane (2:1, v:v) and collected in a new glass tube. The resulting organic phases were individually mixed with 1250 µL of dichloromethane and 2250 µL of ultrapure water to promote phase separation after centrifugation at 568×*g* for 10 min at room temperature. The lipid extracts were then recovered from the organic lower phases, dried under a nitrogen stream and preserved at – 20 °C for further analysis. The final weight of the total lipid extract was determined by gravimetry.

### Phospholipid quantification

Quantification of phospholipids in the total lipid extracts was performed through the phosphorus assay (Bartlett and Lewis 1970). Total lipids were dissolved in 300 µL of dichloromethane and 10 µL of each sample were transferred to glass tubes and dried under a nitrogen stream. Then, 125 µL of 70% perchloric acid was added followed by an incubation at 180 °C for 60 min in a heating block. After cooling at room temperature, 825 µL of MiliQ water, 125 µL of 2.5% aqueous solution of ammonium molybdate [(NH_4_)_6_ Mo_7_O_24_·4H_2_O] and 125 µL of 10% ascorbic acid were added to the samples. Standards were prepared using 0.1–2 µg of phosphate (standard solution of NaH_2_PO_4_·2H_2_O, 100 µg mL^−1^) to perform a standard curve of phosphate and followed the same treatment as the samples. Both samples and standards were incubated at 100 °C in a water bath (Precisterm, JP Selecta) for 10 min. The absorbance of samples and standards was measured at 797 nm using a microplate ultraviolet–visible spectrophotometer (Multiskan GO, Thermo Scientific). The amount of phospholipids present in the samples was estimated by multiplying the phosphorus amount of each sample by 25, the conversion factor between phosphorus and phospholipids (Chapman 1980).

### Hydrophilic interaction liquid chromatography–mass spectrometry

Lipid extracts were analysed by hydrophilic interaction liquid chromatography–mass spectrometry (HILIC–LC–MS) on an Ultimate 3000 Dionex ultrahigh-performance liquid chromatography (UHPLC) system (Thermo Fisher Scientific) with an autosampler coupled online to a Q-Exactive mass spectrometer with Orbitrap^®^ technology (Thermo Fisher, Scientific) (Rey et al. 2023). The analysis was performed using a two-mobile phase solvent system: mobile phase A was a mix of water, acetonitrile and methanol (25/50/25, v:v:v) and mobile phase B contained acetonitrile and methanol (60/40, v:v). Both mobile phases A and B presented ammonium acetate (5 mM). The lipid extracts were dissolved in dichloromethane to obtain a concentration of phospholipid of 1 µg μL^−1^, then a volume corresponding to 10 µg of phospholipid was mixed with 82 µL of the starting eluent (95/5, mobile phase B/mobile phase A, v:v) and 8 µL of phospholipid standards mix (dMPC 0.04 μg, dMPE 0.04 μg, LPC 0.04 μg, dMPG 0.024 μg, dMPS 0.08 μg, tCL (14:0) 0.16 μg, SM (d18:1/17:0) 0.04 μg, Cer (d18:1/17:0) 0.08 μg, dMPA 0.16 μg) and injected into an Ascentis Express HILIC 10 cm × 2.1 mm × 2.7 µm (Supelco, Sigma-Aldrich) at a flow rate of 200 µL min^−1^ and a temperature of 35 °C. Initially, 5% of mobile phase A was held isocratically for 2 min, followed by a linear increase to 48% of mobile phase A within 8 min and a new linear increase to 65% within 5 min, maintained for a period of 2 min, returned to the initial conditions in 3 min and being held for an additional 10 min.

The mass spectrometer employed was operated using positive/negative switching toggles between the positive (electrospray voltage 3.0 kV) and negative (electrospray voltage − 2.7 kV) ion modes with a capillary temperature of 350 °C and a sheath gas flow of 20 U. In MS experiments, a high resolution of 70,000 was used, as well as an automatic gain control (AGC) target of 1 × 10^6^. In tandem mass spectrometry (MS/MS) experiments, a resolution of 17,500 and AGC target of 1 × 10^5^ were used and cycles consisted of one full-scan mass spectrum and ten data-dependent MS/MS scans were repeated continuously throughout the experiments with the dynamic exclusion of 60 s and an intensity threshold of 2 × 10^4^. Normalized collision energy ™ (CE) ranged between 25, 30 and 35 eV. Data acquisition was performed using the Xcalibur data system (V3.3, Thermo Fisher Scientific, USA).

### Data analysis

Polar lipid species were identified through their typical retention time, *m*/*z* mass accuracy (Qual Browser) of ions observed in the LC–MS (≤ 5 ppm) and interpretation of LC–MS and MS/MS spectra to confirm the characteristic fragmentation of the polar head group and fatty acyl chains (Rey et al. 2022). Ion peak integration and assignments were performed using MZmine version 2.53 (Pluskal et al. 2010), based on an in-house lipid database. During data processing, all peaks with raw intensity less than 1e4 were excluded. Integrated peak areas of each lipid species were exported and data were normalized by dividing peak areas from extracted ion chromatograms (XIC) for each lipid species by the peak area of selected internal standard for lipid classes as follows: PC with dMPC; LPC with LPC; PE, LPE, DGTS, MGTS, PE-Cer and CAEP with dMPE; CL with tCL; PI, LPI, PG, LPG, SQDG, SQMG and DGGA with dMPG; PS and LPS with dMPS; MGDG, MGMG, DGDG, DGMG, HexCer and Cer with Cer (d18:1/17:0).

### Statistical analysis

Two-way ANOVAs were performed to compare sea slug DW, relative abundance of lipids and relative abundance of phospholipids. These statistical analyses were performed using R version 4.1.0 (R Core Team 2021) in RStudio 2023.6.1.524 (Posit Team 2023), as well as the chord diagrams which were obtained with the circlize package (Gu et al. 2014).

Normalized XIC areas of the total lipid species identified in the lipidome of *E. crispata* were filtered with the relative standard deviation (RSD = SD/mean, percentage to filter out: 10%) to remove variables that showed low repeatability from the subsequent analysis (Hackstadt and Hess 2009) and datasets were log transformed. Principal component analysis (PCA) was used to identify the major dimensions in the data. A PCA for total lipid species, as well as for each lipid categories dataset, was also performed to visualize the differences between samples from different habitats (Veracruz and Mahahual) and under different feeding conditions (fed and starved). Hierarchical clustering heatmaps were used to better visualize differences between groups. Heatmaps were performed using Euclidean distance measure and the Ward clustering algorithm. The top 50 molecular lipid species were ranked using an ANOVA test. Student's* t* tests were performed to assess the existence of significant differences (*p* value < 0.05) in the log-transformed normalized XIC areas of total lipid species between samples from different habitats, but in the same feeding condition (i.e. Veracruz-Fed vs. Mahahual-Fed, and Veracruz-Starved vs. Mahahual-Starved) and between samples submitted to different feeding conditions (fed vs. 1 week starved) from the same habitat (i.e. Veracruz-Fed vs. Veracruz-Starved, and Mahahual-Fed vs. Mahahual-Starved). Next, *p* values were corrected for multiple testing using Benjamini–Hochberg false discovery rate (FDR, *q* values). All data analysis was performed using Metaboanalyst 5.0 (Pang et al. 2021).

All experimental data are shown as mean ± standard deviation of seven biological replicates (*n* = 7).

## Results

### Lipidome and lipid categories

The highest DW was observed in fed specimens of *E. crispata* originating from Veracruz; however, no significant differences in DW were detected between sampling habitats and feeding conditions (DW: Veracruz-Fed 68.6 ± 19.0 mg; Veracruz-Starved: 43.1 ± 10.1 mg; Mahahual-Fed 47.4 ± 22.4 mg; Mahahual-Starved 44.9 ± 23.2 mg; Two-way ANOVA, habitat *p* value = 0.20, feeding condition *p* value = 0.07, habitat × feeding condition *p* value = 0.13). No significant differences were identified in the relative abundance of lipids by DW (percentage of lipids: Veracruz-Fed 4.2 ± 1.0%; Veracruz-Starved: 6.2 ± 1.4%; Mahahual-Fed 5.9 ± 2.8%; Mahahual-Starved 5.0 ± 2.0%; two-way ANOVA, habitat *p* value = 0.75, feeding condition *p* value = 0.47, habitat × feeding condition *p* value = 0.05) and in the relative abundance of phospholipids present in the total lipid extract (phospholipids: Veracruz-Fed 23.2 ± 5.8%; Veracruz-Starved: 22.9 ± 2.7%; Mahahual-Fed 23.1 ± 4.5%; Mahahual-Starved 24.0 ± 6.0%; two-way ANOVA, habitat *p *value = 0.79, feeding condition *p *value = 0.88, habitat × feeding condition *p* value = 0.76).

The analysis of the polar lipidome of *E. crispata* identified a total of 24 lipid classes by HILIC–LC–MS (Supplementary Table S1) distributed between phospholipids, glycolipids, sphingolipids and betaine lipids. The phospholipid classes identified were phosphatidylcholine (PC, 89 lipid species) and its lyso form (LPC, 22 lipid species), phosphatidylethanolamine (PE, 49 lipid species), and its lyso form (LPE, 18 lipid species), phosphatidylglycerol (PG, 13 lipid species) and its lyso form (LPG, 7 lipid species) phosphatidylinositol (PI, 26 lipid species) and its lyso form (LPI, 6 lipid species), phosphatidylserine (PS, 10 lipid species) and its lyso form (LPS, 1 lipid species), and cardiolipin (CL, 12 lipid species). The glycolipids classes identified were digalactosyl diacylglycerol (DGDG, 19 lipid species) and its lyso form (DGMG, 6 lipid species), monogalactosyl diacylglycerol (MGDG, 30 lipid species) and its lyso form (MGMG, 11 lipid species), diacylglyceryl glucuronide (DGGA, 6 lipid species), and sulphoquinovosyl diacylglycerol (SQDG, 26 lipid species) and its lyso form (SQMG, 2 lipid species). The sphingolipids classes identified were ceramide aminoethylphosphonate (CAEP, 27 lipid species), ceramide phosphoethanolamine (PE-Cer, 7 lipid species), ceramide (Cer, 1 lipid species) and hexosylceramide (HexCer, 7 lipid species). Two classes of betaine lipids were identified diacylglycerol-trimethyl homoserine (DGTS, 44 lipid species) and its lyso form (MGTS, 19 lipid species). A total of 458 polar lipid species (253 phospholipids, 100 glycolipids, 42 sphingolipids, 63 betaine lipids) were identified and quantified in the lipidome of *E. crispata* (Supplementary Table S1).

**Fig. 2:**
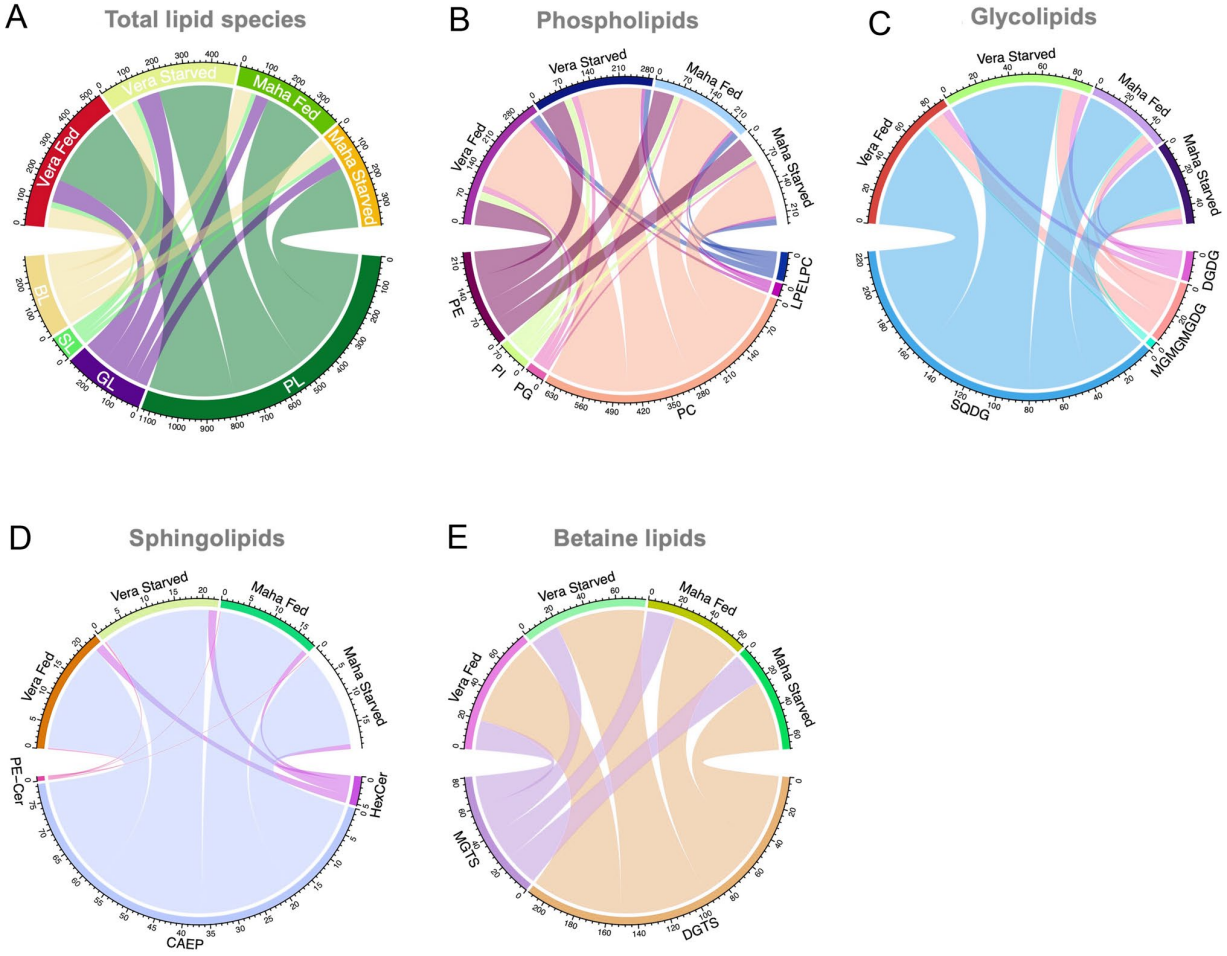
Chord diagrams showing the flows of normalized extracted-ion chromatogram (XIC) areas of **A** total lipids, and the main classes of **B** phospholipids (PL), **C** glycolipids (GL), **D** sphingolipids (SL) and **E** betaine lipids (BL) identified in *Elysia crispata* collected in two different habitats, Veracruz (Vera) and Mahahual (Maha) and under two different feeding conditions, fed and 1 week of starvation. XIC displays the signal intensity of mass-to-charge (*m*/*z*) values which were normalized by the XIC area of the corresponding internal standard

**Fig. 3:**
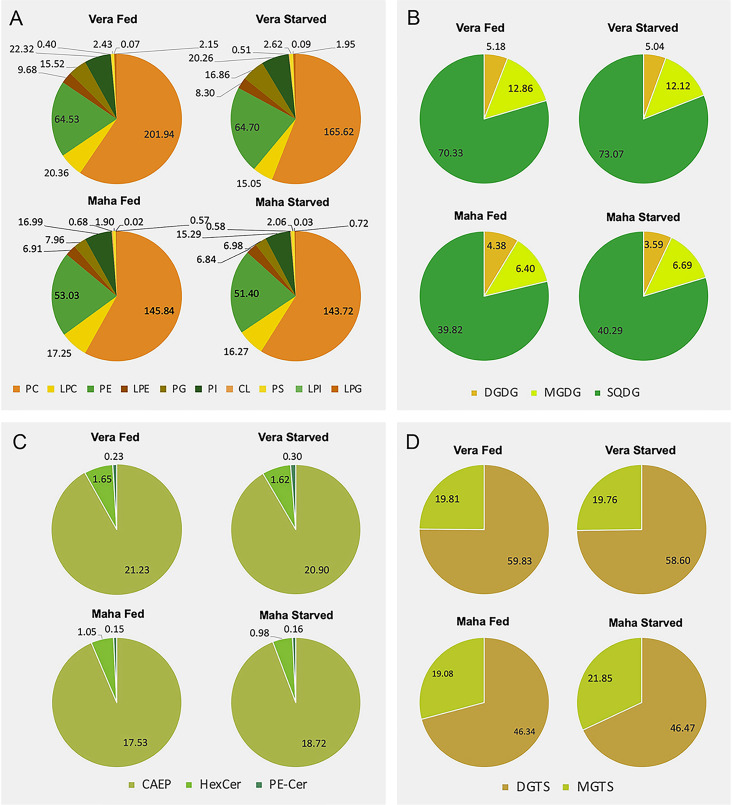
Polar lipidome profile. **A** Phospholipid, **B** glycolipid, **C** sphingolipid and **D** betaine lipids classes identified in *Elysia crispata* collected in two different habitats, Veracruz (Vera) and Mahahual (Maha) and under two different feeding conditions, fed and 1 week of starvation. Data represent sum of normalized extracted-ion chromatogram (XIC) areas for each lipid class

The comparative analysis of normalized XIC areas showed that the polar lipidome of *E. crispata* samples from Veracruz had a higher normalized XIC area in phospholipids and glycolipids than conspecifics from Mahahual (Fig. 2A). The phospholipid classes PC and PE were the most abundant phospholipids, being more abundant in Veracruz than Mahahual (Fig. 2B). The main glycolipid classes recorded (SQDG and MGDG) showed a similar trend, with a higher normalized XIC area in Veracruz than in Mahahual samples (Fig. 2C); the same was also true for sphingolipids (Fig. 2D) and DGTS (Fig. 2E). The distribution of the different polar lipid classes by the different lipid categories is shown in Fig. 3.

**Fig. 4:**
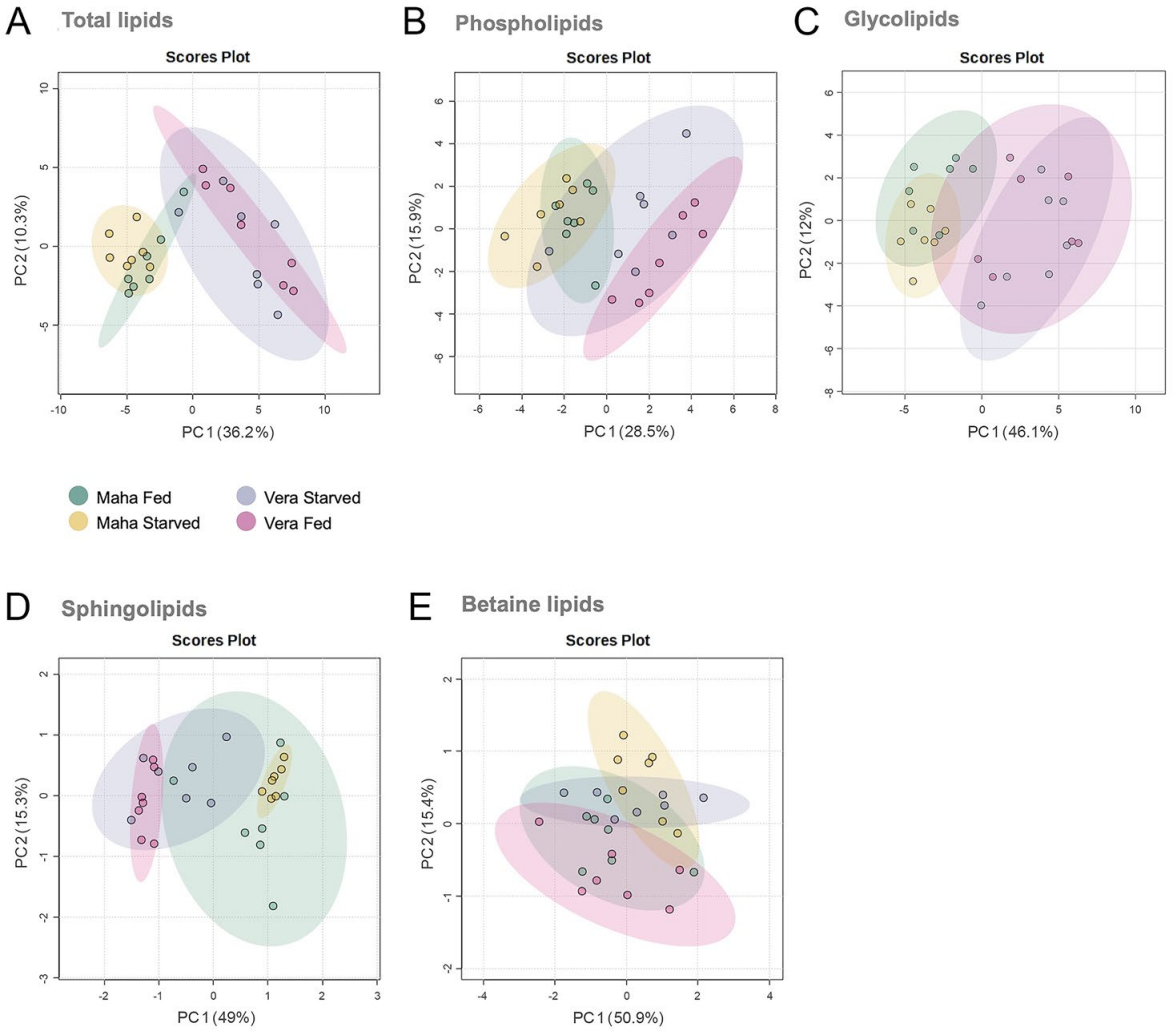
Principal component analysis (PCA) of log-transformed normalized extracted-ion chromatogram (XIC) areas of **A** total lipid species, B phospholipids, **C** glycolipids, **D** sphingolipids and **E** betaine lipids identified in *Elysia crispata* collected in two different habitats, Veracruz (Vera) and Mahahual (Maha) and under two different feeding conditions, fed and 1 week of starvation

The first and second axes of the PCA performed on the total lipid species explained 46.5% of variance (PCA-1: 36.2%, PCA-2: 10.3%) (Fig. 4A). The differences between sampling habitats mostly lined up with the first axis of the PCA. The PCA performed for phospholipids explained 44.4% of variance (PCA-1: 28.5%, PCA-2: 15.9%) (Fig. 4B), while that for glycolipids explained 58.1% of variance (PCA-1: 46.1%, PCA-2: 12.0%) (Fig. 4C). Separation by sampling habitat is clear on the first PCA axis. The PCA of sphingolipids explained 64.3% of variance (PCA-1: 49.0%, PCA-2: 15.3%) (Fig. 4D), with that for betaine lipids explaining 66.3% of variance (PCA-1: 50.9%, PCA-2: 15.4%) (Fig. 4E). An overlap of samples from the same habitat, but with different feeding conditions was observed in the PCA of total lipids and glycolipids.

**Fig. 5:**
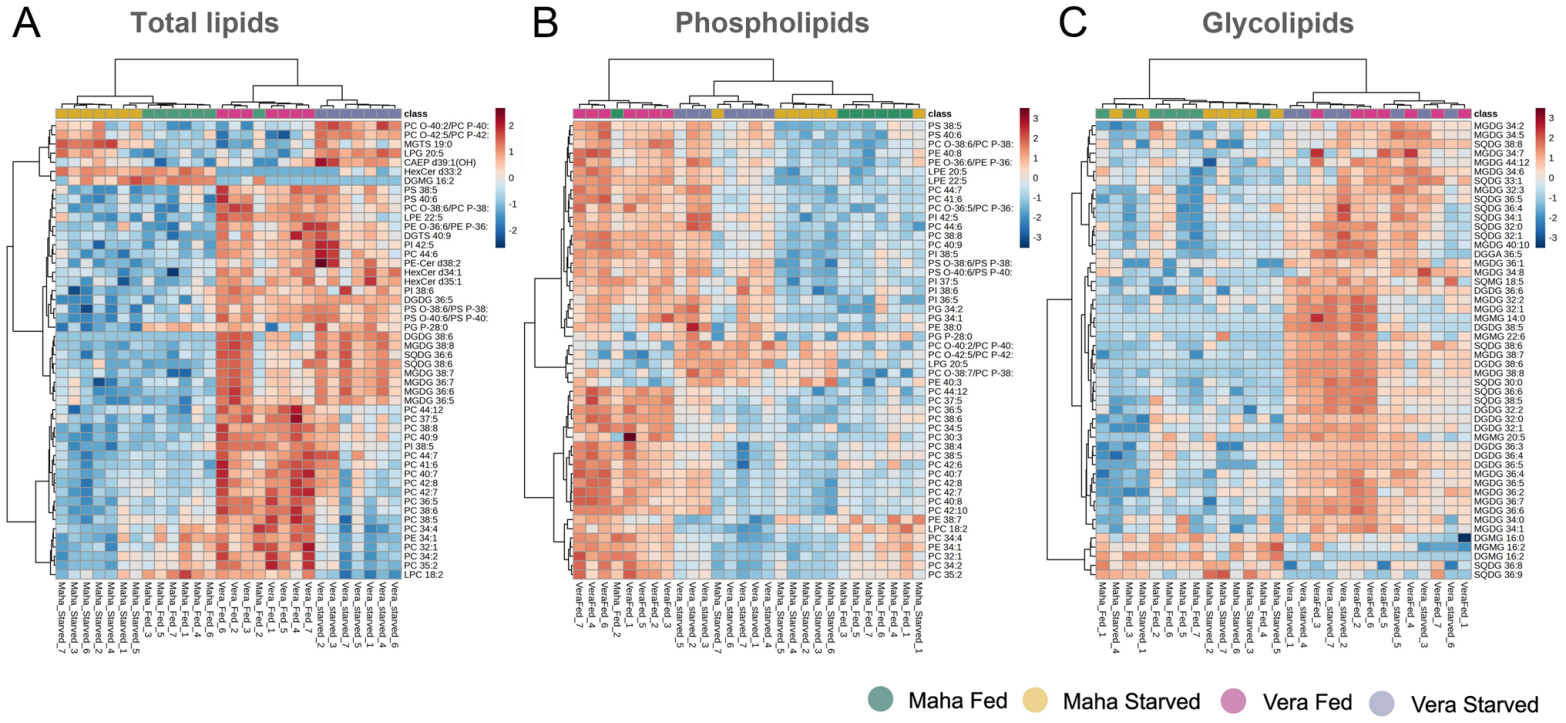
Heatmap/clustering analysis showing the top 50 most significant lipid species in **A** total lipid species, **B** phospholipids and **C** glycolipids discriminating *Elysia crispata* collected in two different habitats, Veracruz (Vera) and Mahahual (Maha) and under two different feeding conditions, fed and 1 week of starvation

The heatmap of the total polar lipidome using the top 50 molecular lipid species ranked using an ANOVA test showed a separation between sampling groups and conditions (Fig. 5A). Phospholipid and glycolipid molecular species contributed most to discrimination between samples (the top 50 most significant lipid species in the ANOVA test included 33 phospholipids, 10 glycolipids, 5 sphingolipids and 2 betaine lipids). The heatmap of the top 50 phospholipid species, ranked using an ANOVA test, showed a separation between two clusters, one cluster included all samples from Veracruz-Fed and one sample of Mahahual-Fed. Another cluster with three subclusters mostly separated starved samples from Veracruz, Mahahual-Starved and Mahahual-Fed (Fig. 5B). The phospholipids that contributed the most to the discrimination between groups belonged to the PC class. The heatmap of the top 50 glycolipids grouped samples according to sampling habitat, but did not account for whether these were from fed or starved specimens (Fig. 5C). The glycolipids that most contributed to the separation were MGDG and SQDG. The heatmap of the sphingolipids showed a cluster separation by habitat (Supplementary Fig. S1a), a pattern also evidenced by glycolipid heatmap. The heatmap of the top 50 betaine lipid species randomly grouped samples, with no clear pattern being perceptible (Supplementary Fig. S1b).

### Lipidome variability in sampling habitats (Mahahual-Fed vs. Veracruz-Fed and Mahahual-Starved vs. Veracruz-Starved)

**Fig. 6:**
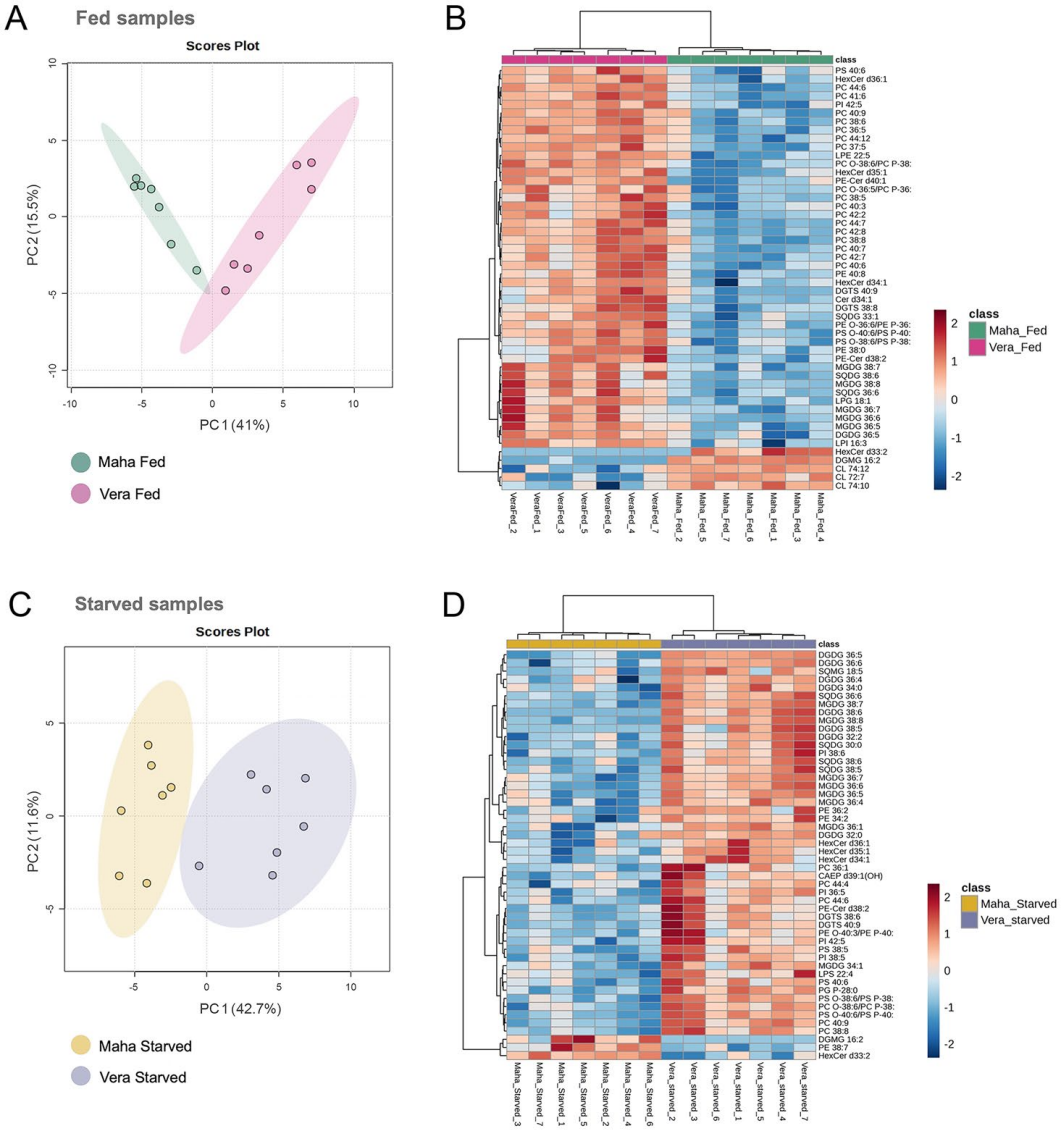
**A** Principal component analysis (PCA) of log-transformed normalized extracted-ion chromatogram (XIC) areas and **B** heatmap/clustering analysis (PCA) showing the top 50 most significant lipid species discriminating *Elysia crispata* sampled in two different habitats Veracruz (Vera) and Mahahual (Maha) under fed conditions. **C** PCA of log-transformed XIC areas and **D** heatmap/clustering analysis showing the top 50 most significant lipid species discriminating *E. crispata* sampled in two different habitats Veracruz (Vera) and Mahahual (Maha) and starved for a week

The PCA of total lipids identified in the polar lipidome of *E. crispata* under fed condition but from different habitats explained 56.5% of variance (PCA-1: 41.0%, PCA-2: 15.5%), with a separation between habitats shown on the first axis (Fig. 6A). A cluster separation by habitat can also be observed on the heatmap performed using the top 50 most significant lipid species (Fig. 6B). The lipid species that most contributed to the separation between habitats included 94 phospholipids, 31 glycolipids, 13 sphingolipids and 8 betaine lipids (Supplementary Table S2).

The PCA of total lipid species identified in the polar lipidome sampled from different habitats explained 54.3% of variance (PCA-1: 42.7%, PCA-2: 11.6%) (Fig. 6C), with a clear cluster separation by habitat being observed in the heatmap of the top 50 most significant lipid species (Fig. 6D). The lipid species that most contributed to the separation between habitats included 41 glycolipids, 34 phospholipids, 10 sphingolipids and two betaine lipids (Supplementary Table S3).

### Lipidome variability between feeding condition (Veracruz-Fed vs. Veracruz-Starved and Mahahual-Fed vs. Mahahual-Starved)

**Fig. 7:**
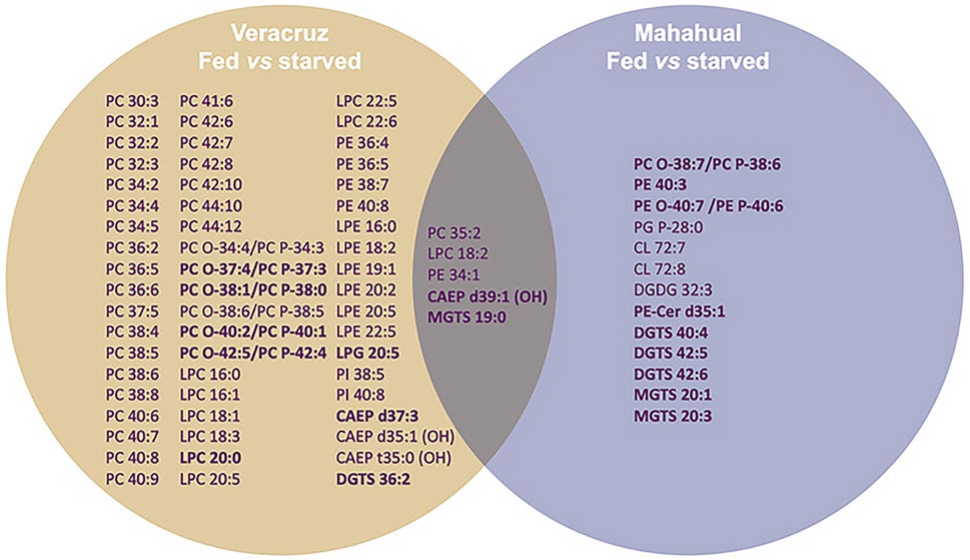
Molecular species that showed significant differences in the comparison between fed versus starved *Elysia crispata* sampled in two different habitats, Veracruz (fed vs. starved) and Mahahual (fed vs. starved), (Student’s *t* test, *p* < 0.05, with FDR correction). The lipid species that significantly incremented under starved conditions were marked in bold

To better understand the influence of putatively different dietary sources in the polar lipidome of *E. crispata* under starved conditions, a comparative analysis with samples from the same habitat, but subjected to different feeding conditions was performed. The comparison of the XIC areas between fed and starved samples within the same habitat (i.e. Vera-Fed vs. Vera-Starved and Maha-Fed vs. Maha-Starved) showed that of the lipid species identified, only 62 in samples from Veracruz and 18 in samples from Mahahual had significant differences between feeding conditions (fed vs. starved) (Supplementary Table S4). In the Veracruz samples, 56 phospholipids, four sphingolipids and two betaine lipids showed significant differences between fed and starved specimens, while in the Mahahual samples these differences were found in nine phospholipid, six betaine lipids, two sphingolipids and one glycolipid (Supplementary Table S5). Only five of these lipid species (i.e. PC 35:2; LPC 18:2; PE 34:1; CAEP d39:1(OH); MGTS 19:0) were significantly different between feeding conditions in both sampling habitats, showing the same trend in both (Fig. 7). Most of the molecular species that significantly changed between fed versus starved specimens decreased under starvation. The few molecular species that increased under starvation were mostly ether phospholipids, sphingolipids (i.e. CAEP and PE-Cer) or betaine lipids (i.e. DGTS and MGTS) (Fig. 7). The lipid category that showed the greatest significant differences between fed and starved conditions were phospholipids.

## Discussion

### Mass spectrometry highlights polar lipid diversity in a photosynthetic animal

The loss of DW after 1 week of starvation was more pronounced in the Veracruz samples compared to those from Mahahual, potentially reflecting their different nutritional states, although no significant differences were recorded between feeding conditions and habitats. A recent study examining changes in the relative weight of *E. crispata* under different feeding regimes with the macroalga *Bryopsis plumosa* demonstrated that individuals fed regularly experienced high weight loss during the first starvation days (~ 16 days) compared to those with less frequent feeding regimens (i.e. once per week or every 2 weeks). However, after 45 days, specimens subjected to irregular feeding exhibited the highest loss of relative weight (Cartaxana et al. 2023). This study demonstrated that specimens in a good nutritional state experienced a significant impact on their weight in the initial days; however, they exhibited high resilience after reaching the inflection point. The loss of DW in specimens from Veracruz suggests that they were likely in better nutritional condition than those from Mahahual, which experienced a greater impact from starvation during the initial days.

Most of the studied photosynthetic sea slugs are stenophagous, feeding and retaining functional chloroplasts from a specific genus or species of siphonaceous green macroalgae (Baumgartner and Toth 2014; Maeda et al. 2012). *Elysia crispata* is known to feed on a high diversity of macroalgae species (see supplementary Table S1 in Vital et al. 2021). The present results on the polar lipidome of *E. crispata* suggest that its diet varies across its geographic distribution areas and the habitat conditions, providing specimens with different abilities to cope with periods of food scarcity.

Lipids are a very diverse molecular class with different biological functions that can be used as biomarkers in marine environments (Řezanka et al. 2018; Rey et al. 2022; Edwards 2023). As such, comparative lipidomic studies can elucidate the metabolic and physiological state of marine organisms (Rey et al. 2020; Sikorskaya et al. 2020; Tang et al. 2018). The main lipid classes identified in the present work have been described in previous lipidomics studies of *Elysia viridis, Placida dendritica* and *Elysia timida* (Rey et al. 2020, 2023). The characterization of *E. crispata* lipidome allowed the identification of new lipid classes that have not been reported in previous studies of sea slugs, such as the phospholipid CL, the glycolipid DGGA or the sphingolipid HexCer. Nonetheless, it should be highlighted that the fact that these lipid classes have not been identified in previous studies does not necessarily imply their absence in the previously studied sea slugs.

The sample clustering of phospholipids clearly separated fed individuals from Veracruz from those under other conditions. The high content of PC with esterified PUFA (e.g. PC 18:3_20:5; PC 20:2_22:6; PC 20:2_24:4) in fed specimens from Veracruz may be related to their dietary resources. In sacoglossan sea slugs, lipids may have been directly derived from dietary intake, through translocation of metabolites from kleptoplasts to the animal cells (Cruz et al. 2020; Cartaxana et al. 2021) or from digestion of kleptoplasts (Laetz et al. 2017a, b; Laetz and Wägele 2017). A lipidomic study of nudibranch molluscs demonstrated that dietary fatty acids were used to assemble PE, PC and PS, while the configuration of PI and CAEP was not influenced by the diet (Imbs and Grigorchuk 2019). The study of Cartaxana et al. (2021) of the sacoglossan *E. timida* using marked ^13^C-labelling, demonstrated an increment in the levels of ^13^C-labelling in the elongation states of the PUFA 20:5 *n*-3 (eicosapentaenoic acid, EPA) to 22:5 *n*-3 and from 20:4 *n*-6 (arachidonic acid; ARA) to 22:4 *n*-6 under light conditions. This suggests that the synthesis of some PUFA in sea slugs’ tissues is promoted by the photosynthetic activity of kleptoplasts (Cartaxana et al. 2021).

In eukaryotic cells, mitochondria are involved in a range of metabolic and bioenergetic processes that are vital for cell survival and aging (Blier et al. 2017; Paradies et al. 2019). CL are synthesized in the inner mitochondrial membrane, representing a major phospholipid in this membrane. This lipid class plays important roles in mitochondria, such as in membrane morphology, stability and dynamics, biogenesis and protein import, as well as mitophagy and apoptosis (Paradies et al. 2019). CL are involved in the induction of apoptosis through interaction with cytochrome c (Blier et al. 2017). The peroxidation activity of cytochrome c on polyunsaturated fatty acyl groups of CL promote the migration of oxidized CL to the outer mitochondrial membrane and the release of pro-apoptotic factors into the cytosol (Blier et al. 2017). Autophagy levels have shown a notable increase in the sea slug *E. viridis* when subjected to dark conditions (Frankenbach et al. 2023), suggesting that more sea slug tissue would be required to compensate for the lost energy demands due to the lack of photosynthesis.

The main functions of PI are cell signalling, membrane traffic and motility (Marat and Haucke 2016), although their role has been poorly studied in marine invertebrates. Thermal stress in the soft coral *Sinularia* sp. promoted a decrease of the amount of PI (Sikorskaya et al. 2020). The decrease of PI observed in sea slugs sampled in Mahahual suggests that these individuals were subjected to stress.

The presence of glycolipids and betaine lipids in the lipidome of *E. crispata* is related to the retention of chloroplasts in their epithelial cells (Rey et al. 2017). The comparison of glycolipid molecular species allowed a clear separation between habitats, a separation that was not observed in the clustering analysis of betaine lipid species. The heatmap for betaine lipids randomly grouped samples from different experimental groups, indicating a lack of habitat-based differentiation.

The glycolipids that contributed most to the separation between habitats were all more expressed in Veracruz, except for SQDG 36:9, SQDG 36:8, DGMG 16:2, MGMG 16:2 and DGMG 16:0, which were more expressed in Mahahual. The increment of lyso glycolipids (i.e. DGMG and MGMG) is related to the action of galactolipases (Lion et al. 2006). Chloroplast lipid hydrolysis can be activated under stress conditions, as a defence mechanism (Lion et al. 2006), or to release lipid-mediated signalling molecules responsible for the activation of several metabolomic pathways (Hyun et al. 2008; Okazaki and Saito 2014). The clustering analysis of glycolipids demonstrated a clear separation by habitats rather than feeding conditions, which is probably related to the sources of chloroplasts in the different sampling locations. It is also worth noting that their abundance was not modified after 1 week of starvation. The elevated content of glycolipids and PG in sea slugs from Veracruz may be related to a higher content of chloroplasts in the animal cells, suggesting that *E. crispata* from Mahahual may have suffered periods of food shortage.

### Lipidome variability is influenced by habitat conditions (Mahahual-Fed vs. Veracruz-Fed and Mahahual-Starved vs. Veracruz-Starved)

As sampling months were different at each locality (March in Mahahual and June in Veracruz), effects related to sampling period cannot be completely excluded (Carrillo et al. 2007), although the two regions have different macroalgae compositions (Garduño-Solórzano et al. 2005; Palomino-Alvarez et al. 2021). This was supported by our results, as the comparison of samples under the same condition but from different habitats confirms that the lipid species analysed promoted a higher variability in specimens from Veracruz than from Mahahual. The high level of intra-specific variability in Veracruz samples must be related to a greater diversity of food sources that is reflected in several components of the sea slug lipidome. The lipids that contributed the most to the separation between habitats in fed samples were phospholipids. However, in the comparison of samples under starved conditions, glycolipids contributed most to the separation between locations. These results may be explained by the presence of different chloroplast sources or varying abundances within the sea slug tissues across the two habitats studied. The main food source of sacoglossan sea slugs are macroalgae, although a few sacoglossans can feed on diatoms, sea grasses and sea slug eggs (Jensen 1980). A study comparing the polar lipidome of several macroalgae species from different phyla demonstrated a species-specific clustering, suggesting a distinctiveness of the profile of lipid molecules (Lopes et al. 2021). Fed *E. crispata* samples provide a snapshot of sea slug lipidome featuring contributions from the latest food sources they have ingested; alternatively, starved specimens reflect the lipidome of the chloroplasts incorporated into the sea slug tissues. Glycolipids identified in specimens under starvation are characteristic of the chloroplasts previously sequestered by the animal cells (Rey et al. 2017). Samples of starved sea slugs from Veracruz showed a high abundance of glycolipids, suggesting a high amount of kleptoplats and a better nutritional state and possibly a higher diversity of available food resources.

### Different habitats provide different strategies to cope with periods of food shortage (Veracruz-Fed vs. Veracruz-Starved and Mahahual-Fed vs. Mahahual-Starved)

The comparison of polar lipids between feeding conditions in the same sampling site showed that the biggest differences between fed and starved specimens were observed in Veracruz (62 lipid species significantly different between fed and starved). These differences are associated with a more diverse and richer source of suitable dietary items in Veracruz rather than in Mahahual. The polar lipidome of the Mahahual samples was remained stable during 1 week of starvation (only 18 lipid species were significantly different between fed and starved *E. crispata*). Interestingly, the lipid species identified as significantly different between fed and starved states are not characteristic of algae tissues, except for some DGTS, MGTS and one DGDG. These results suggest that 1 week of starvation is insufficient to initiate the degradation of plastid membranes in *E. crispata*. This photosynthetic sea slug has demonstrated the ability to maintain functional kleptoplasts from the macroalgae *B. plumosa* and *Penicillus capitatus* for over 10 weeks (Curtis et al. 2015; Middlebrooks et al. 2011). Therefore, plastid functionality and the longevity of kleptoplasts appear to be dependent on the origin of the plastids (Cartaxana et al. 2023).

The few changes recorded in the polar lipidome of both fed and starved sea slugs from Mahahual suggest a potential adaptation of these sea slugs to periods of food shortage experienced prior to sampling. The lipid species that increased under starvation were ether phospholipids, sphingolipids (CAEP and PE-Cer) or betaine lipids (DGTS and MGTS). Ether phospholipids, including the alkenylacyl phospholipids, known as plasmalogen, are important membrane components of cell organelles in marine invertebrates (Imbs et al. 2021; Imbs and Grigorchuk 2019; Imbs and Velansky 2021), including the nucleus, endoplasmic reticulum or mitochondria. They play important roles in cell membranes as structural components (Paul et al. 2019), protective molecules to combat the oxidative stress to maintain oxidative stability (Broniec et al. 2011; Wang and Wang 2010) and signal transduction (Braverman and Moser 2012). Ether phospholipids exhibit distinct physical properties essential for membrane fusion, a characteristic that also supports their role in facilitating membrane trafficking processes (Dean and Lodhi 2018). Their abundance in starved sea slugs must therefore be related to a protective role that these biomolecules may play in cell membranes when having to cope with starvation.

## Conclusions

The examination of the polar lipidome of *E. crispata,* sourced from different habitats and under different feeding conditions, has revealed significant variations across its geographic distribution, suggesting different amounts of retained chloroplasts or dietary sources. Notably, the lipidome of *E. crispata* is highly sensitive to different habitats and the consequences of starvation periods are influenced by preceding nutritional states.

Samples collected from Veracruz exhibited a singular lipidome profile, characterized by a higher abundance of most lipid classes, even after a week of starvation. This suggests a better nutritional state and a more diverse diet in this region. Conversely, individuals from Mahahual displayed a lipidome composition indicative of a poorer diet or even periods of starvation before collection. Additionally, lack of habitat-based differentiation in the clustering analysis of betaine lipids suggests that the role of these lipids in kleptoplasty requires further investigation.

The findings from the present study underscore the relevance of using lipidomics as a valuable tool for ecological studies of marine invertebrates, shedding light on the intricate relationships between habitat conditions, dietary patterns and physiological responses.

## Supplementary Information

Below is the link to the electronic supplementary material.

Supplementary file1 (DOCX 1583 KB)

Supplementary file2 (XLSX 167 KB)

Supplementary file3 (DOCX 39 KB)

Supplementary file4 (DOCX 28 KB)

Supplementary file5 (DOCX 24 KB)

Supplementary file6 (DOCX 17 KB)

## Data Availability

All data supporting the findings of this study are available within the paper and its Supplementary Information.
