# Supplementary material for: Habitat shapes the lipidome of the tropical photosynthetic sea slug Elysia crispata
Source: Mar Life Sci Technol. 2025 Apr 7;7(2):382–96. doi: 10.1007/s42995-025-00281-1 (PMC12102446; doi:10.1007/s42995-025-00281-1)
Supplement: Supplementary file 1 — Supplementary file1 (DOCX 1583 KB) [file 42995_2025_281_MOESM1_ESM.docx]

**Supplementary Figure S1:** Heatmap/clustering analysis showing the most significant lipid species in a) sphingolipid and e) betaine lipid species identified in *Elysia crispata* collected in two different habitats, Veracruz (Vera) and Mahahual (Maha) and under two different feeding conditions, fed and one week of starvation.
