# Supplementary material for: Habitat shapes the lipidome of the tropical photosynthetic sea slug Elysia crispata
Source: Mar Life Sci Technol. 2025 Apr 7;7(2):382–96. doi: 10.1007/s42995-025-00281-1 (PMC12102446; doi:10.1007/s42995-025-00281-1)
Supplement: Supplementary file 3 — Supplementary file3 (DOCX 39 KB) [file 42995_2025_281_MOESM3_ESM.docx]

**Supplementary Table S2** Results of Student’s *t* test (log transformed normalized extracted-ion chromatogram (XIC) areas) of polar lipid molecular species identified in samples of *Elysia crispata* from two different habitats (Veracruz and Mahahual) and under fed conditions. Adjustment of p-values for multiple comparisons was performed using Benjamini–Hochberg correction for the false discovery rate (FDR).

| Lipid species | Lipid category | t.stat | p.value | -10log(p) | FDR |
| --- | --- | --- | --- | --- | --- |
| DGMG 16:2 | Glycolipid | 15.024 | 3.82E-09 | 8.4185 | 1.57E-06 |
| PS O-40:6/PS P-40:5 | Phospholipid | -8.7854 | 1.42E-06 | 5.8467 | 0.00029323 |
| PC 41:6 | Phospholipid | -8.1833 | 2.98E-06 | 5.5259 | 0.00040915 |
| PC 44:6 | Phospholipid | -7.7725 | 5.05E-06 | 5.2971 | 0.00051967 |
| PE-Cer d40:1 | Sphingolipid | -7.3008 | 9.47E-06 | 5.0238 | 0.00073242 |
| PC 42:8 | Phospholipid | -7.2136 | 1.07E-05 | 4.972 | 0.00073242 |
| PC O-38:6/PC P-38:5 | Phospholipid | -6.93 | 1.58E-05 | 4.8007 | 0.0007797 |
| HexCer d35:1 | Sphingolipid | -6.8574 | 1.75E-05 | 4.756 | 0.0007797 |
| PC 44:7 | Phospholipid | -6.8397 | 1.80E-05 | 4.7451 | 0.0007797 |
| LPG 18:1 | Phospholipid | -6.8039 | 1.89E-05 | 4.723 | 0.0007797 |
| PC 36:5 | Phospholipid | -6.6818 | 2.25E-05 | 4.647 | 0.00084438 |
| PC 37:5 | Phospholipid | -6.5042 | 2.92E-05 | 4.5349 | 0.00096841 |
| PC 38:6 | Phospholipid | -6.4729 | 3.06E-05 | 4.5149 | 0.00096841 |
| PC 42:2 | Phospholipid | -6.0411 | 5.84E-05 | 4.2338 | 0.0017178 |
| SQDG 38:6 | Glycolipid | -5.9809 | 6.40E-05 | 4.1937 | 0.0017585 |
| SQDG 33:1 | Glycolipid | -5.8706 | 7.59E-05 | 4.1197 | 0.0019548 |
| PC 42:7 | Phospholipid | -5.7606 | 9.01E-05 | 4.0451 | 0.0021843 |
| SQDG 36:6 | Glycolipid | -5.6486 | 0.00010752 | 3.9685 | 0.0023542 |
| PC 38:8 | Phospholipid | -5.62 | 0.0001125 | 3.9488 | 0.0023542 |
| PE O-36:6/PE P-36:5 | Phospholipid | -5.5818 | 0.00011955 | 3.9225 | 0.0023542 |
| MGDG 38:8 | Glycolipid | -5.561 | 0.00012359 | 3.908 | 0.0023542 |
| MGDG 38:7 | Glycolipid | -5.5102 | 0.00013405 | 3.8727 | 0.0023542 |
| LPE 22:5 | Phospholipid | -5.4943 | 0.00013751 | 3.8617 | 0.0023542 |
| PC 40:7 | Phospholipid | -5.4674 | 0.00014359 | 3.8429 | 0.0023542 |
| HexCer d36:1 | Sphingolipid | -5.4582 | 0.00014572 | 3.8365 | 0.0023542 |
| PC 40:9 | Phospholipid | -5.4462 | 0.00014856 | 3.8281 | 0.0023542 |
| PI 42:5 | Phospholipid | -5.4164 | 0.00015588 | 3.8072 | 0.0023745 |
| PC 44:12 | Phospholipid | -5.395 | 0.00016137 | 3.7922 | 0.0023745 |
| PE-Cer d38:2 | Sphingolipid | -5.2428 | 0.00020682 | 3.6844 | 0.0029383 |
| PC 40:6 | Phospholipid | -5.2107 | 0.00021804 | 3.6615 | 0.0029944 |
| MGDG 36:6 | Glycolipid | -5.1708 | 0.00023283 | 3.633 | 0.0030944 |
| Cer d34:1 | Sphingolipid | -5.0394 | 0.00028961 | 3.5382 | 0.0035336 |
| DGTS 40:9 | Betaine lipid | -5.0258 | 0.00029624 | 3.5284 | 0.0035336 |
| HexCer d34:1 | Sphingolipid | -5.0153 | 0.00030149 | 3.5207 | 0.0035336 |
| HexCer d33:2 | Sphingolipid | 5.0138 | 0.00030224 | 3.5197 | 0.0035336 |
| PE 40:8 | Phospholipid | -5.0011 | 0.00030876 | 3.5104 | 0.0035336 |
| PS 40:6 | Phospholipid | -4.9715 | 0.00032447 | 3.4888 | 0.0035975 |
| DGTS 38:8 | Betaine lipid | -4.9581 | 0.00033181 | 3.4791 | 0.0035975 |
| MGDG 36:7 | Glycolipid | -4.8511 | 0.00039752 | 3.4006 | 0.0041994 |
| DGDG 36:5 | Glycolipid | -4.7919 | 0.00043957 | 3.357 | 0.004502 |
| MGDG 36:5 | Glycolipid | -4.7727 | 0.00045417 | 3.3428 | 0.004502 |
| PC 38:5 | Phospholipid | -4.7666 | 0.00045894 | 3.3382 | 0.004502 |
| CL 74:12 | Phospholipid | 4.7492 | 0.0004728 | 3.3253 | 0.0045301 |
| PE 38:0 | Phospholipid | -4.7237 | 0.0004938 | 3.3065 | 0.0046237 |
| LPI 16:3 | Phospholipid | -4.6662 | 0.00054501 | 3.2636 | 0.0049899 |
| CL 74:10 | Phospholipid | 4.5952 | 0.00061607 | 3.2104 | 0.0055178 |
| PC 40:3 | Phospholipid | -4.5575 | 0.00065762 | 3.182 | 0.0057647 |
| PC O-36:5/PC P-36:4 | Phospholipid | -4.5182 | 0.00070406 | 3.1524 | 0.0059864 |
| PS O-38:6/PS P-38:5 | Phospholipid | -4.5117 | 0.00071197 | 3.1475 | 0.0059864 |
| CL 72:7 | Phospholipid | 4.4409 | 0.00080562 | 3.0939 | 0.0066383 |
| LPG 18:0 | Phospholipid | -4.4194 | 0.00083645 | 3.0776 | 0.0067572 |
| PS 38:5 | Phospholipid | -4.4039 | 0.00085952 | 3.0657 | 0.0068101 |
| PE O-40:6/PE P-40:5 | Phospholipid | -4.3797 | 0.00089676 | 3.0473 | 0.006971 |
| LPE 20:5 | Phospholipid | -4.3601 | 0.00092816 | 3.0324 | 0.0070815 |
| PC 44:8 | Phospholipid | -4.342 | 0.0009583 | 3.0185 | 0.0071785 |
| DGDG 38:6 | Glycolipid | -4.2888 | 0.0010525 | 2.9778 | 0.0077431 |
| CL 72:8 | Phospholipid | 4.2646 | 0.0010985 | 2.9592 | 0.0077611 |
| LPG 18:2 | Phospholipid | -4.2583 | 0.0011109 | 2.9543 | 0.0077611 |
| PI 38:5 | Phospholipid | -4.258 | 0.0011114 | 2.9541 | 0.0077611 |
| PC 34:5 | Phospholipid | -4.2281 | 0.0011718 | 2.9311 | 0.0080467 |
| PE-Cer d36:1 | Sphingolipid | -4.1934 | 0.0012465 | 2.9043 | 0.0083069 |
| CL 74:11 | Phospholipid | 4.1918 | 0.0012501 | 2.9031 | 0.0083069 |
| PC 30:3 | Phospholipid | -4.164 | 0.0013135 | 2.8816 | 0.0085898 |
| MGDG 34:8 | Glycolipid | -4.1519 | 0.0013421 | 2.8722 | 0.0086399 |
| SQMG 18:5 | Glycolipid | -4.1318 | 0.001391 | 2.8567 | 0.0087486 |
| PC 40:8 | Phospholipid | -4.1276 | 0.0014015 | 2.8534 | 0.0087486 |
| PC 44:5 | Phospholipid | -4.1098 | 0.0014468 | 2.8396 | 0.008897 |
| DGDG 36:6 | Glycolipid | -4.081 | 0.0015234 | 2.8172 | 0.0091055 |
| SQDG 36:5 | Glycolipid | -4.073 | 0.0015452 | 2.811 | 0.0091055 |
| CL 72:9 | Phospholipid | 4.0724 | 0.0015471 | 2.8105 | 0.0091055 |
| PI 37:5 | Phospholipid | -4.0231 | 0.00169 | 2.7721 | 0.0097859 |
| PC 40:4 | Phospholipid | -4.0165 | 0.0017102 | 2.767 | 0.0097859 |
| PC 40:2 | Phospholipid | -3.9824 | 0.0018184 | 2.7403 | 0.010263 |
| PC 42:10 | Phospholipid | -3.9535 | 0.0019157 | 2.7177 | 0.010666 |
| LPE 19:1 | Phospholipid | -3.9213 | 0.0020305 | 2.6924 | 0.011154 |
| PC 38:4 | Phospholipid | -3.9116 | 0.0020663 | 2.6848 | 0.011202 |
| LPI 18:0 | Phospholipid | -3.8696 | 0.0022294 | 2.6518 | 0.011929 |
| SQDG 30:0 | Glycolipid | -3.8346 | 0.0023755 | 2.6243 | 0.012547 |
| DGTS 38:6 | Betaine lipid | -3.801 | 0.002525 | 2.5977 | 0.013114 |
| PC 35:2 | Phospholipid | -3.7964 | 0.0025464 | 2.5941 | 0.013114 |
| LPE 20:1 | Phospholipid | -3.726 | 0.0028948 | 2.5384 | 0.014724 |
| MGDG 40:10 | Glycolipid | -3.71 | 0.0029809 | 2.5257 | 0.014977 |
| PI O-40:4/PI P-40:3 | Phospholipid | 3.6857 | 0.0031161 | 2.5064 | 0.015324 |
| PG 34:1 | Phospholipid | -3.6801 | 0.0031484 | 2.5019 | 0.015324 |
| DGTS 37:1 | Betaine lipid | 3.6778 | 0.0031614 | 2.5001 | 0.015324 |
| CAEP t36:0 | Sphingolipid | -3.5489 | 0.0040047 | 2.3974 | 0.019153 |
| PE O-38:6/PE P38:5 | Phospholipid | -3.5435 | 0.0040445 | 2.3931 | 0.019153 |
| MGDG 34:6 | Glycolipid | -3.5043 | 0.0043477 | 2.3617 | 0.020355 |
| PG 34:2 | Phospholipid | -3.494 | 0.0044309 | 2.3535 | 0.020512 |
| LPE 20:2 | Phospholipid | -3.4843 | 0.0045105 | 2.3458 | 0.020648 |
| DGTS 42:8 | Betaine lipid | -3.4472 | 0.0048297 | 2.3161 | 0.021866 |
| MGDG 32:3 | Glycolipid | -3.4395 | 0.004899 | 2.3099 | 0.021939 |
| PC O-38:5/PC P-38:4 | Phospholipid | -3.4236 | 0.0050446 | 2.2972 | 0.022348 |
| LPI 20:1 | Phospholipid | -3.4043 | 0.0052284 | 2.2816 | 0.022916 |
| PE O-39:6/PE P-39:5 | Phospholipid | -3.3868 | 0.0053996 | 2.2676 | 0.023247 |
| PC 34:0 | Phospholipid | -3.3851 | 0.0054169 | 2.2662 | 0.023247 |
| LPE 20:3 | Phospholipid | -3.3384 | 0.0059052 | 2.2288 | 0.025082 |
| PS O-40:2/PS P-40:1 | Phospholipid | -3.328 | 0.0060201 | 2.2204 | 0.025309 |
| PC 34:2 | Phospholipid | -3.2953 | 0.0063962 | 2.1941 | 0.026572 |
| DGDG 32:1 | Glycolipid | -3.2908 | 0.0064495 | 2.1905 | 0.026572 |
| LPC 16:0 | Phospholipid | -3.2769 | 0.0066175 | 2.1793 | 0.026994 |
| PC 42:6 | Phospholipid | -3.2609 | 0.0068168 | 2.1664 | 0.027534 |
| PE-Cer d38:3 | Sphingolipid | -3.2177 | 0.0073854 | 2.1316 | 0.029542 |
| PG 40:3 | Phospholipid | -3.2045 | 0.0075692 | 2.1209 | 0.029903 |
| DGTS 36:2 | Betaine lipid | 3.2008 | 0.007621 | 2.118 | 0.029903 |
| SQDG 32:1 | Glycolipid | -3.1953 | 0.0076996 | 2.1135 | 0.029927 |
| PC 36:1 | Phospholipid | -3.1898 | 0.0077784 | 2.1091 | 0.02995 |
| CAEP d35:1 (OH) | Sphingolipid | -3.1683 | 0.008095 | 2.0918 | 0.030612 |
| PI 36:5 | Phospholipid | -3.168 | 0.0080989 | 2.0916 | 0.030612 |
| CL 72:10 | Phospholipid | 3.1492 | 0.0083877 | 2.0764 | 0.031416 |
| DGGA 36:1 | Glycolipid | -3.0977 | 0.0092299 | 2.0348 | 0.034259 |
| LPS 22:4 | Phospholipid | -3.0759 | 0.0096117 | 2.0172 | 0.035357 |
| MGDG 36:4 | Glycolipid | -3.0496 | 0.010092 | 1.996 | 0.036795 |
| PC 38:7 | Phospholipid | -3.0447 | 0.010184 | 1.9921 | 0.036806 |
| SQDG 34:4 | Glycolipid | -3.0157 | 0.010748 | 1.9687 | 0.038245 |
| PC 38:1 | Phospholipid | -3.0147 | 0.010768 | 1.9679 | 0.038245 |
| MGMG 22:6 | Glycolipid | -2.9997 | 0.011072 | 1.9558 | 0.038462 |
| LPE 18:1 | Phospholipid | -2.9896 | 0.011282 | 1.9476 | 0.038462 |
| PG 34:4 | Phospholipid | -2.9882 | 0.011311 | 1.9465 | 0.038462 |
| MGTS 22:6 | Betaine lipid | -2.9864 | 0.011349 | 1.945 | 0.038462 |
| DGGA 38:4 | Glycolipid | -2.9861 | 0.011357 | 1.9447 | 0.038462 |
| MGDG 36:2 | Glycolipid | -2.9845 | 0.011389 | 1.9435 | 0.038462 |
| PC 40:10 | Phospholipid | -2.9792 | 0.011503 | 1.9392 | 0.038531 |
| PC 38:2 | Phospholipid | -2.9492 | 0.012161 | 1.915 | 0.040186 |
| PI 38:6 | Phospholipid | -2.9479 | 0.012192 | 1.9139 | 0.040186 |
| PI O-36:5/PI P-36:4 | Phospholipid | -2.9278 | 0.012656 | 1.8977 | 0.041384 |
| MGDG 32:1 | Glycolipid | -2.9191 | 0.012862 | 1.8907 | 0.041725 |
| PC 44:4 | Phospholipid | -2.8924 | 0.013515 | 1.8692 | 0.043503 |
| SQDG 38:5 | Glycolipid | -2.8838 | 0.013734 | 1.8622 | 0.043833 |
| CL 76:12 | Phospholipid | 2.88 | 0.013831 | 1.8592 | 0.043833 |
| DGTS 38:7 | Betaine lipid | -2.8754 | 0.013949 | 1.8554 | 0.043871 |
| PC 36:4 | Phospholipid | -2.8598 | 0.014359 | 1.8429 | 0.044816 |
| PE O-40:3/PE P-40:2 | Phospholipid | -2.8545 | 0.0145 | 1.8386 | 0.044915 |
| LPC 18:3 | Phospholipid | -2.849 | 0.014649 | 1.8342 | 0.044915 |
| DGDG 36:4 | Glycolipid | -2.8465 | 0.014717 | 1.8322 | 0.044915 |
| PI 38:5 (OH) | Phospholipid | -2.8309 | 0.015152 | 1.8195 | 0.045901 |
| PC O-38:7/PC P-38:6 | Phospholipid | -2.8177 | 0.015527 | 1.8089 | 0.046695 |
| CAEP d39:1(OH) | Sphingolipid | -2.809 | 0.015778 | 1.8019 | 0.046727 |
| PC 42:3 | Phospholipid | -2.8088 | 0.015784 | 1.8018 | 0.046727 |
| MGDG 32:2 | Glycolipid | -2.8056 | 0.015878 | 1.7992 | 0.046727 |
| CAEP d39:1 | Sphingolipid | -2.7993 | 0.016064 | 1.7941 | 0.046939 |
| LPC 14:0 | Phospholipid | -2.7865 | 0.016449 | 1.7838 | 0.047727 |
| CL 76:11 | Phospholipid | 2.7668 | 0.017062 | 1.768 | 0.04892 |
| LPE O-16:0 | Phospholipid | -2.7622 | 0.01721 | 1.7642 | 0.04892 |
| CL 72:11 | Phospholipid | 2.7619 | 0.017217 | 1.764 | 0.04892 |
| LPE 20:4 | Phospholipid | -2.7481 | 0.017666 | 1.7529 | 0.049852 |
