# Supplementary material for: Habitat shapes the lipidome of the tropical photosynthetic sea slug Elysia crispata
Source: Mar Life Sci Technol. 2025 Apr 7;7(2):382–96. doi: 10.1007/s42995-025-00281-1 (PMC12102446; doi:10.1007/s42995-025-00281-1)
Supplement: Supplementary file 4 — Supplementary file4 (DOCX 28 KB) [file 42995_2025_281_MOESM4_ESM.docx]

**Supplementary Table S3** Results of Student’s *t* test (log transformed normalized extracted-ion chromatogram (XIC) areas) of polar lipid molecular species identified in samples of *Elysia crispata* from two different habitats (Veracruz and Mahahual) and under starved conditions. Adjustment of p-values for multiple comparisons was performed using Benjamini–Hochberg correction for the false discovery rate (FDR).

| Lipid species | Lipid category | t.stat | p.value | -10log(p) | FDR |
| --- | --- | --- | --- | --- | --- |
| MGDG 38:8 | Glycolipid | -11.152 | 1.09E-07 | 6.9627 | 4.49E-05 |
| MGDG 38:7 | Glycolipid | -9.758 | 4.66E-07 | 6.3313 | 9.61E-05 |
| DGDG 36:5 | Glycolipid | -8.7947 | 1.41E-06 | 5.8515 | 0.00016907 |
| PS O-40:6/PS P-40:5 | Phospholipid | -8.5383 | 1.92E-06 | 5.717 | 0.00016907 |
| DGDG 38:6 | Glycolipid | -8.4834 | 2.05E-06 | 5.6879 | 0.00016907 |
| PC 40:9 | Phospholipid | -7.1285 | 1.20E-05 | 4.921 | 0.00082359 |
| PG P-28:0 | Phospholipid | -6.9585 | 1.52E-05 | 4.8181 | 0.0008574 |
| SQDG 36:6 | Glycolipid | -6.8941 | 1.66E-05 | 4.7786 | 0.0008574 |
| MGDG 36:6 | Glycolipid | -6.4691 | 3.07E-05 | 4.5125 | 0.0014067 |
| PS O-38:6/PS P-38:5 | Phospholipid | -6.3548 | 3.64E-05 | 4.4391 | 0.0014945 |
| SQDG 38:6 | Glycolipid | -6.2929 | 3.99E-05 | 4.399 | 0.0014945 |
| DGDG 36:6 | Glycolipid | -5.9497 | 6.72E-05 | 4.1729 | 0.0023059 |
| PC 38:8 | Phospholipid | -5.5211 | 0.00013172 | 3.8803 | 0.0041747 |
| MGDG 36:7 | Glycolipid | -5.4182 | 0.00015544 | 3.8084 | 0.0045743 |
| DGTS 40:9 | Betaine lipid | -5.352 | 0.00017305 | 3.7618 | 0.004753 |
| MGDG 36:5 | Glycolipid | -5.2624 | 0.0002003 | 3.6983 | 0.0048813 |
| SQDG 38:5 | Glycolipid | -5.236 | 0.00020914 | 3.6796 | 0.0048813 |
| PI 38:5 | Phospholipid | -5.2241 | 0.00021326 | 3.6711 | 0.0048813 |
| SQDG 30:0 | Glycolipid | -5.1388 | 0.0002455 | 3.6099 | 0.0051252 |
| HexCer d33:2 | Sphingolipid | 5.1307 | 0.0002488 | 3.6042 | 0.0051252 |
| PI 36:5 | Phospholipid | -5.0683 | 0.00027596 | 3.5592 | 0.005414 |
| PI 38:6 | Phospholipid | -5.0238 | 0.00029723 | 3.5269 | 0.0055662 |
| PC O-38:6/PC P-38:5 | Phospholipid | -4.9579 | 0.00033195 | 3.4789 | 0.0059462 |
| SQDG 32:0 | Glycolipid | -4.7006 | 0.00051375 | 3.2893 | 0.0088193 |
| PE-Cer d38:2 | Sphingolipid | -4.651 | 0.00055953 | 3.2522 | 0.009221 |
| DGTS 38:6 | Betaine lipid | -4.5788 | 0.00063372 | 3.1981 | 0.0093372 |
| PS 40:6 | Phospholipid | -4.5698 | 0.00064368 | 3.1913 | 0.0093372 |
| PS 38:5 | Phospholipid | -4.5173 | 0.00070516 | 3.1517 | 0.0093372 |
| HexCer d35:1 | Sphingolipid | -4.514 | 0.00070913 | 3.1493 | 0.0093372 |
| DGDG 38:5 | Glycolipid | -4.5036 | 0.00072214 | 3.1414 | 0.0093372 |
| SQDG 36:9 | Glycolipid | 4.5005 | 0.00072604 | 3.139 | 0.0093372 |
| DGDG 32:2 | Glycolipid | -4.4889 | 0.00074083 | 3.1303 | 0.0093372 |
| HexCer d34:1 | Sphingolipid | -4.4835 | 0.00074788 | 3.1262 | 0.0093372 |
| PG 34:1 | Phospholipid | -4.3281 | 0.00098205 | 3.0079 | 0.011567 |
| PE 38:7 | Phospholipid | 4.3147 | 0.0010056 | 2.9976 | 0.011567 |
| PI 42:5 | Phospholipid | -4.3118 | 0.0010107 | 2.9954 | 0.011567 |
| SQMG 18:5 | Glycolipid | -4.2823 | 0.0010647 | 2.9728 | 0.011639 |
| PG 34:2 | Phospholipid | -4.2668 | 0.0010943 | 2.9609 | 0.011639 |
| MGDG 36:1 | Glycolipid | -4.263 | 0.0011017 | 2.9579 | 0.011639 |
| DGDG 34:0 | Glycolipid | -4.2213 | 0.0011862 | 2.9259 | 0.012113 |
| DGDG 32:0 | Glycolipid | -4.2044 | 0.0012222 | 2.9129 | 0.012113 |
| MGDG 34:1 | Glycolipid | -4.1987 | 0.0012348 | 2.9084 | 0.012113 |
| DGMG 16:2 | Glycolipid | 4.1428 | 0.0013641 | 2.8652 | 0.01307 |
| PE-Cer d40:2 | Sphingolipid | -4.0695 | 0.0015549 | 2.8083 | 0.01456 |
| SQDG 38:8 | Glycolipid | -4.0494 | 0.001612 | 2.7926 | 0.014758 |
| SQDG 36:5 | Glycolipid | -4.0242 | 0.0016867 | 2.773 | 0.015107 |
| PC 44:6 | Phospholipid | -3.9726 | 0.0018508 | 2.7326 | 0.016224 |
| PE-Cer d40:3 | Sphingolipid | -3.942 | 0.0019559 | 2.7086 | 0.016788 |
| MGDG 36:4 | Glycolipid | -3.9045 | 0.002093 | 2.6792 | 0.01745 |
| HexCer d36:1 | Sphingolipid | -3.8909 | 0.0021453 | 2.6685 | 0.01745 |
| DGDG 32:1 | Glycolipid | -3.8727 | 0.002217 | 2.6542 | 0.01745 |
| MGDG 32:3 | Glycolipid | -3.8567 | 0.0022822 | 2.6416 | 0.01745 |
| LPS 22:4 | Phospholipid | -3.8491 | 0.0023138 | 2.6357 | 0.01745 |
| CAEP d39:1(OH) | Sphingolipid | -3.8392 | 0.0023559 | 2.6278 | 0.01745 |
| PE 36:2 | Phospholipid | -3.8337 | 0.0023795 | 2.6235 | 0.01745 |
| PI 37:5 | Phospholipid | -3.8264 | 0.0024112 | 2.6178 | 0.01745 |
| PC O-40:6/PC P-40:5 | Phospholipid | -3.8213 | 0.0024337 | 2.6137 | 0.01745 |
| SQDG 32:1 | Glycolipid | -3.8162 | 0.0024565 | 2.6097 | 0.01745 |
| DGDG 36:4 | Glycolipid | -3.8056 | 0.0025041 | 2.6013 | 0.017487 |
| MGDG 32:1 | Glycolipid | -3.755 | 0.0027459 | 2.5613 | 0.018855 |
| PC 44:4 | Phospholipid | -3.699 | 0.0030414 | 2.5169 | 0.020542 |
| MGDG 34:5 | Glycolipid | -3.5693 | 0.0038577 | 2.4137 | 0.025635 |
| MGMG 14:0 | Glycolipid | -3.5533 | 0.0039726 | 2.4009 | 0.02598 |
| MGDG 34:8 | Glycolipid | -3.4884 | 0.0044762 | 2.3491 | 0.028816 |
| PE 34:2 | Phospholipid | -3.4655 | 0.0046692 | 2.3308 | 0.029306 |
| PE-Cer d36:1 | Sphingolipid | -3.4626 | 0.0046946 | 2.3284 | 0.029306 |
| PC 36:1 | Phospholipid | -3.4023 | 0.0052478 | 2.28 | 0.032178 |
| PE O-40:3/PE P-40:2 | Phospholipid | -3.3958 | 0.0053109 | 2.2748 | 0.032178 |
| PE O-36:6/PE P-36:5 | Phospholipid | -3.3757 | 0.0055116 | 2.2587 | 0.03291 |
| PC 44:7 | Phospholipid | -3.3526 | 0.0057524 | 2.2402 | 0.033756 |
| PC 32:0 | Phospholipid | -3.3465 | 0.0058172 | 2.2353 | 0.033756 |
| MGDG 36:2 | Glycolipid | -3.3244 | 0.0060602 | 2.2175 | 0.034285 |
| PE 38:0 | Phospholipid | -3.3231 | 0.0060748 | 2.2165 | 0.034285 |
| CAEP t36:0 | Sphingolipid | -3.2871 | 0.0064945 | 2.1875 | 0.036159 |
| PG 34:0 | Phospholipid | -3.2688 | 0.0067177 | 2.1728 | 0.036903 |
| MGMG 16:0 | Glycolipid | -3.228 | 0.0072458 | 2.1399 | 0.03928 |
| PC O-36:5/PC P-36:4 | Phospholipid | -3.2183 | 0.0073781 | 2.1321 | 0.039478 |
| SQDG 36:8 | Glycolipid | 3.2066 | 0.0075402 | 2.1226 | 0.039828 |
| PC 41:6 | Phospholipid | -3.179 | 0.0079358 | 2.1004 | 0.041387 |
| PE O-40:6/PE P-40:5 | Phospholipid | -3.1355 | 0.0086042 | 2.0653 | 0.044312 |
| MGDG 34:0 | Glycolipid | -3.0957 | 0.009263 | 2.0332 | 0.046591 |
| MGMG 16:2 | Glycolipid | 3.0952 | 0.009273 | 2.0328 | 0.046591 |
| MGDG 34:2 | Glycolipid | -3.0797 | 0.009544 | 2.0203 | 0.047375 |
| LPC 18:2 | Phospholipid | 3.0432 | 0.010213 | 1.9908 | 0.049803 |
| DGGA 36:5 | Glycolipid | -3.0382 | 0.010309 | 1.9868 | 0.049803 |
| PC 44:5 | Phospholipid | -3.0327 | 0.010415 | 1.9823 | 0.049803 |
| MGDG 32:2 | Glycolipid | -3.0274 | 0.010517 | 1.9781 | 0.049803 |
