# Supplementary material for: Habitat shapes the lipidome of the tropical photosynthetic sea slug Elysia crispata
Source: Mar Life Sci Technol. 2025 Apr 7;7(2):382–96. doi: 10.1007/s42995-025-00281-1 (PMC12102446; doi:10.1007/s42995-025-00281-1)
Supplement: Supplementary file 5 — Supplementary file5 (DOCX 24 KB) [file 42995_2025_281_MOESM5_ESM.docx]

**Supplementary Table S4** Results of Student’s *t* test (log transformed normalized extracted-ion chromatogram (XIC) areas) of polar lipid molecular species identified in samples of *Elysia crispata* from Veracruz and under two different feeding conditions (fed versus starved). Adjustment of p-values for multiple comparisons was performed using Benjamini–Hochberg correction for the false discovery rate (FDR).

| Lipid species | Lipid category | t.stat | p.value | -10log(p) | FDR |
| --- | --- | --- | --- | --- | --- |
| PC 38:6 | Phospholipid | -8.0937 | 3.34E-06 | 5.4767 | 0.00071672 |
| LPC 18:2 | Phospholipid | -8.0608 | 3.48E-06 | 5.4585 | 0.00071672 |
| PC 34:5 | Phospholipid | -7.655 | 5.89E-06 | 5.2301 | 0.00077431 |
| PC 44:12 | Phospholipid | -7.4713 | 7.52E-06 | 5.1239 | 0.00077431 |
| PE 34:1 | Phospholipid | -7.1921 | 1.10E-05 | 4.9592 | 0.00090523 |
| PC O-40:2/PC P-40:1 | Phospholipid | 6.8079 | 1.88E-05 | 4.7254 | 0.0012448 |
| PC 36:5 | Phospholipid | -6.649 | 2.36E-05 | 4.6264 | 0.0012448 |
| PC 34:2 | Phospholipid | -6.6336 | 2.42E-05 | 4.6167 | 0.0012448 |
| PC 34:4 | Phospholipid | -6.5421 | 2.76E-05 | 4.559 | 0.0012639 |
| LPG 20:5 | Phospholipid | 6.4515 | 3.15E-05 | 4.5012 | 0.0012993 |
| PC 35:2 | Phospholipid | -6.2715 | 4.12E-05 | 4.3851 | 0.0014209 |
| PC O-42:5/PC P-42:4 | Phospholipid | 6.2684 | 4.14E-05 | 4.3831 | 0.0014209 |
| PC 32:1 | Phospholipid | -5.6376 | 0.00010941 | 3.9609 | 0.0034214 |
| PC 38:5 | Phospholipid | -5.5993 | 0.00011626 | 3.9346 | 0.0034214 |
| PE 38:7 | Phospholipid | -5.3544 | 0.00017238 | 3.7635 | 0.0047347 |
| PI 38:5 | Phospholipid | -5.1271 | 0.00025031 | 3.6015 | 0.0064454 |
| PC 44:10 | Phospholipid | -4.919 | 0.00035442 | 3.4505 | 0.0085895 |
| PC 32:2 | Phospholipid | -4.845 | 0.00040162 | 3.3962 | 0.0091926 |
| CAEP d37:3 | Sphingolipid | 4.7829 | 0.00044633 | 3.3503 | 0.0096783 |
| LPC 18:3 | Phospholipid | -4.5555 | 0.00065991 | 3.1805 | 0.013594 |
| PC 40:9 | Phospholipid | -4.5026 | 0.00072338 | 3.1406 | 0.014192 |
| PC 42:6 | Phospholipid | -4.4483 | 0.00079529 | 3.0995 | 0.014894 |
| LPE 22:5 | Phospholipid | -4.3714 | 0.00090998 | 3.041 | 0.016301 |
| PC 37:5 | Phospholipid | -4.343 | 0.00095654 | 3.0193 | 0.016421 |
| LPC 16:1 | Phospholipid | -4.1885 | 0.0012574 | 2.9005 | 0.020593 |
| PC 42:7 | Phospholipid | -4.1699 | 0.0012996 | 2.8862 | 0.020593 |
| PC 32:3 | Phospholipid | -4.0251 | 0.001684 | 2.7737 | 0.025696 |
| PC 40:6 | Phospholipid | -3.9654 | 0.0018751 | 2.727 | 0.027359 |
| LPC 22:5 | Phospholipid | -3.9506 | 0.0019258 | 2.7154 | 0.027359 |
| PC 30:3 | Phospholipid | -3.8297 | 0.002397 | 2.6203 | 0.031955 |
| PC 42:8 | Phospholipid | -3.828 | 0.0024044 | 2.619 | 0.031955 |
| LPE 16:0 | Phospholipid | -3.7369 | 0.002838 | 2.547 | 0.034659 |
| MGTS 19:0 | Betaine Lipid | 3.7365 | 0.0028402 | 2.5467 | 0.034659 |
| PE 36:4 | Phospholipid | -3.7326 | 0.0028602 | 2.5436 | 0.034659 |
| PC 38:8 | Phospholipid | -3.7098 | 0.0029819 | 2.5255 | 0.035101 |
| PC 40:7 | Phospholipid | -3.6823 | 0.0031357 | 2.5037 | 0.035125 |
| PC 38:4 | Phospholipid | -3.679 | 0.0031544 | 2.5011 | 0.035125 |
| LPC 18:1 | Phospholipid | -3.6622 | 0.0032531 | 2.4877 | 0.03527 |
| CAEP d35:1 (OH) | Sphingolipid | -3.637 | 0.0034067 | 2.4677 | 0.035988 |
| PC 36:6 | Phospholipid | -3.6131 | 0.0035591 | 2.4487 | 0.036432 |
| PE 40:8 | Phospholipid | -3.591 | 0.0037068 | 2.431 | 0.036432 |
| PC 36:2 | Phospholipid | -3.5835 | 0.0037582 | 2.425 | 0.036432 |
| LPC 20:5 | Phospholipid | -3.564 | 0.003895 | 2.4095 | 0.036432 |
| PC 40:8 | Phospholipid | -3.5426 | 0.0040512 | 2.3924 | 0.036432 |
| LPC 20:0 | Phospholipid | 3.5378 | 0.0040876 | 2.3885 | 0.036432 |
| LPE 20:5 | Phospholipid | -3.524 | 0.0041925 | 2.3775 | 0.036432 |
| LPE 18:2 | Phospholipid | -3.5114 | 0.0042912 | 2.3674 | 0.036432 |
| PC O-38:1/PC P-38:0 | Phospholipid | 3.4965 | 0.0044103 | 2.3555 | 0.036432 |
| PC O-34:4/PC P-34:3 | Phospholipid | -3.4839 | 0.0045138 | 2.3455 | 0.036432 |
| CAEP t35:0 (OH) | Sphingolipid | -3.4836 | 0.0045167 | 2.3452 | 0.036432 |
| LPE 20:2 | Phospholipid | -3.4808 | 0.0045398 | 2.343 | 0.036432 |
| PC 41:6 | Phospholipid | -3.466 | 0.0046651 | 2.3311 | 0.036432 |
| PC 42:10 | Phospholipid | -3.4635 | 0.0046866 | 2.3291 | 0.036432 |
| PI 40:8 | Phospholipid | -3.4248 | 0.0050333 | 2.2981 | 0.038402 |
| LPC 22:6 | Phospholipid | -3.4065 | 0.0052065 | 2.2835 | 0.038505 |
| DGTS 36:2 | Betaine Lipid | 3.4037 | 0.0052337 | 2.2812 | 0.038505 |
| CAEP d39:1(OH) | Sphingolipid | 3.3153 | 0.0061639 | 2.2101 | 0.043677 |
| LPE 19:1 | Phospholipid | -3.3095 | 0.0062306 | 2.2055 | 0.043677 |
| PE 36:5 | Phospholipid | -3.3055 | 0.0062762 | 2.2023 | 0.043677 |
| PC O-37:4/PC P-37:3 | Phospholipid | 3.2983 | 0.0063607 | 2.1965 | 0.043677 |
| PC O-38:6/PC P-38:5 | Phospholipid | -3.2887 | 0.0064747 | 2.1888 | 0.043731 |
| LPC 16:0 | Phospholipid | -3.2141 | 0.0074356 | 2.1287 | 0.049411 |
