# Supplementary material for: Habitat shapes the lipidome of the tropical photosynthetic sea slug Elysia crispata
Source: Mar Life Sci Technol. 2025 Apr 7;7(2):382–96. doi: 10.1007/s42995-025-00281-1 (PMC12102446; doi:10.1007/s42995-025-00281-1)
Supplement: Supplementary file 6 — Supplementary file6 (DOCX 17 KB) [file 42995_2025_281_MOESM6_ESM.docx]

**Supplementary Table S5** Results of Student’s *t* test (log transformed normalized extracted-ion chromatogram (XIC) areas) of polar lipid molecular species identified in samples of *Elysia crispata* from Mahahual and under two different feeding conditions (fed versus starved). Adjustment of p-values for multiple comparisons was performed using Benjamini–Hochberg correction for the false discovery rate (FDR).

| Lipid species | Lipid caterogy | t.stat | p.value | -10log(p) | FDR |
| --- | --- | --- | --- | --- | --- |
| PE O-40:7/PE P-40:6 | Phospholipid | -8.8266 | 1.36E-06 | 5.868 | 0.0005543 |
| PG P-28:0 | Phospholipid | 8.1478 | 3.12E-06 | 5.5064 | 0.00063718 |
| DGTS 40:4 | Betaine Lipid | -6.7872 | 1.94E-05 | 4.7126 | 0.0026423 |
| MGTS 19:0 | Betaine Lipid | -5.6377 | 0.00010939 | 3.961 | 0.011185 |
| PE 40:3 | Phospholipid | -5.3824 | 0.00016472 | 3.7833 | 0.013474 |
| CL 72:8 | Phospholipid | 5.2016 | 0.00022131 | 3.655 | 0.015086 |
| LPC 18:2 | Phospholipid | 4.7837 | 0.00044571 | 3.351 | 0.026042 |
| DGTS 42:6 | Betaine Lipid | -4.575 | 0.00063793 | 3.1952 | 0.02881 |
| PE-Cer d35:1 | Sphingolipid | -4.5535 | 0.00066211 | 3.1791 | 0.02881 |
| PC 35:2 | Phospholipid | 4.5179 | 0.0007044 | 3.1522 | 0.02881 |
| PE 34:1 | Phospholipid | 4.3559 | 0.0009351 | 3.0291 | 0.034352 |
| CL 72:7 | Phospholipid | 4.3133 | 0.0010079 | 2.9966 | 0.034352 |
| PC O-38:7/PC P-38:6 | Phospholipid | -4.2443 | 0.0011388 | 2.9435 | 0.035829 |
| DGDG 32:3 | Glycolipid | 4.1484 | 0.0013504 | 2.8695 | 0.038309 |
| DGTS 42:5 | Betaine Lipid | -4.1262 | 0.001405 | 2.8523 | 0.038309 |
| MGTS 20:3 | Betaine Lipid | -3.9551 | 0.00191 | 2.719 | 0.04429 |
| CAEP d39:1(OH) | Sphingolipid | -3.95 | 0.0019279 | 2.7149 | 0.04429 |
| MGTS 20:1 | Betaine Lipid | -3.9439 | 0.0019492 | 2.7101 | 0.04429 |
